# A comprehensive model for intimate partner violence in South African primary care: action research

**DOI:** 10.1186/1472-6963-12-399

**Published:** 2012-11-14

**Authors:** Kate Joyner, Bob Mash

**Affiliations:** 1 Nursing Division, Faculty of Medicine and Health Sciences, Stellenbosch University, P O Box 19063, Tygerberg 7505, South Africa; 2 Division of Family Medicine and Primary Care, Faculty of Medicine and Health Sciences, Stellenbosch University, Francie Van Zijl Ave, Parow, South Africa

**Keywords:** Interpersonal violence, Intimate partner violence, Domestic violence, Spouse abuse, Mental health, Action research, Co-operative inquiry, Primary care, South Africa, Health services, Health systems

## Abstract

**Background:**

Despite extensive evidence on the magnitude of intimate partner violence (IPV) as a public health problem worldwide, insubstantial progress has been made in the development and implementation of sufficiently comprehensive health services. This study aimed to implement, evaluate and adapt a published protocol for the screening and management of IPV and to recommend a model of care that could be taken to scale in our underdeveloped South African primary health care system.

**Methods:**

Professional action research utilised a co-operative inquiry group that consisted of four nurses, one doctor and a qualitative researcher. The inquiry group implemented the protocol in two urban and three rural primary care facilities. Over a period of 14 months the group reflected on their experience, modified the protocol and developed recommendations on a practical but comprehensive model of care.

**Results:**

The original protocol had to be adapted in terms of its expectations of the primary care providers, overly forensic orientation, lack of depth in terms of mental health, validity of the danger assessment and safety planning process, and need for ongoing empowerment and support. A three-tier model resulted: case finding and clinical care provision by primary care providers; psychological, social and legal assistance by ‘IPV champions’ followed by a group empowerment process; and then ongoing community-based support groups.

**Conclusion:**

The inquiry process led to a model of comprehensive and intersectoral care that is integrated at the facility level and which is now being piloted in the Western Cape, South Africa.

## Background

The South African burden of disease reveals an extremely violent society with the highest reported intimate femicide rate [1,2]. Most South African social contexts are characterised by oppression of women. At a fundamental level, disrespect for the feminine seems validated by cultural norms and values which prioritise males over females in multiple ways. This normative framework impacts negatively on the quality of people’s relationships and their self-expectations. Despite a progressive constitution and legislation, IPV is still regarded as culturally acceptable, and thus, in many contexts, is normalised [3].

The health consequences of intimate partner violence (IPV) can be categorised as fatal and non-fatal [4]. Fatal outcomes include femicide, suicide, maternal mortality, antepartum haemorrhage, abortion, stillbirth and AIDS. Non-fatal consequences comprise burns, fractures, chronic pain syndromes and mental illness, problems with hearing and sight, arthritis, seizures, headaches, sexually transmitted infections (STIs), HIV, and pelvic inflammatory disease. Indirect consequences of IPV include stomach ulcers and other gastrointestinal disturbances, heart disease, hypertension, unwanted pregnancy, low birth weight and premature labor [5]. Indirect health consequences also extend to abusers compromising their partners’ health by withholding medication, changing a prescription, cancelling appointments, or keeping partners awake [6].

The impact on a woman of even a single incident of physical violence in an intimate relationship should not be underestimated. Use of any violence in a relationship can dramatically alter the balance of power, destroying respect, openness and trust and resulting in a permanent sense of inequality, threat and loss [7]. Recent findings of the World Health Organisation’s multi-country study on women’s health and IPV suggest that the mental effects of violence last long after the violent episode. Moreover, cumulative abuse impacts powerfully on health [4].

International research on IPV undertaken within mental health care institutions and settings is scarce. One study set in New Zealand, England and the United States found that over 50% of women traumatised by IPV suffered a psychiatric disorder. Most notable were the elevated rates of mood and eating disorders [8]. Similarly, in an English primary health care context IPV showed a strong association with most mental health conditions, particularly if experienced during the preceding year [9].

Most research has focused on the nature and prevalence of IPV, but relatively little has been published on interventions or models for care. Baldwin-Ragaven testifies to the proliferation of peer-reviewed articles measuring the problem and documenting the consequences of our failure to act, commenting: “For any other disease process as costly in financial and human measures we would demand answers, find cures, and disseminate evidence about interventions. What is it about IPV?” [10]. There is a clear moral argument that health providers should attend to the problematic impact of IPV on health. Women experiencing IPV present to all health care settings, usually without naming the IPV problem. There is evidence that women appreciate inquiry about IPV and can benefit from intervention [11-13].

There are no existing guidelines for the management of IPV in primary care that have been operationalised in the South African setting. Currently IPV is largely unrecognised by primary care providers and in the few cases that are diagnosed the standard of care is fragmented, poorly coordinated, lacking in continuity and missing important aspects. The best attempt to address this issue has been seen in the PC101 guideline that was developed for nurses, where an approach to the abused patient is included under mental health [14]. This guideline is currently being evaluated in the Western Cape. Our study aimed to implement and adapt the first published protocol on screening for and management of IPV in the South African primary care setting and to recommend a model of care for IPV. At the time of this study this was the only protocol that was on offer for testing in clinical practice.

## Methods

### Study design

Professional action research is ideal for innovating alternative health service delivery systems [15]. It is one of four typologies of action research [16]. Professionals work collectively on a problem identified from their practice with the aim of improving practice and conceptualising their learning. Such professional action research closes the gap between theory and practice, while learning remains highly contextualised. A contextually appropriate application of this is the co-operative inquiry group method, which was adapted by Mash and Meulenberg-Buskens to develop medical education materials, and has been the overarching action research methodology utilised in this project [17]. A co-operative inquiry group implemented a published South African protocol for the screening and management of women experiencing IPV [18]. The underlying assumption was that participants in the inquiry group would create new knowledge from their concrete experience; by observing and reflecting thereupon; by forming abstract concepts and generalisations; and by testing the implications of these concepts in new situations. The co-operative inquiry group, which included the authors, worked with the standard four-step action research cycle: planning, action, observation and reflection [17].

### Setting

Primary care is offered through a network of community health centres and clinics. Community health centres provide services through larger multidisciplinary teams in more urban areas and first contact could be with a nurse or a doctor. Health centres may provide a range of services including mental health, family planning, services for STIs and HIV, maternal and child health, orthopaedics, dental and emergency care. Fixed and mobile clinics are smaller nurse-run services and are particularly common in rural areas. At fixed clinics doctors usually consult on a weekly basis and are employed to support the nurses and see more complicated patients.

In order to test the protocol in rural and urban settings, two urban and three rural facilities, serving historically disadvantaged communities free of charge, were purposively selected in the Western Cape. The two urban facilities are health centres, while the three rural facilities comprise one health centre and two smaller clinics. They were selected for being representative of primary care facilities in the region, having mental health and other services as required by the protocol, a sufficient workload to ensure enough participants, and a private room.

Site A is situated in a formerly designated ‘black group area’ and Site B in a former ‘coloured group area’. Both are urban health centres serving economically disadvantaged residents who are reliant on these health services. Each facility serves approximately 400 patients a day. All general primary health care services are provided by a team of health care providers (clinical nurse practitioners and doctors), a social worker and a psychiatric nurse. Both sites offer preventative services in the form of family planning, immunisation, voluntary counselling and testing for HIV and tuberculosis clinics. After-hours services comprise the trauma unit at Site A and the adjacent maternity facility at Site B.

Site C is situated in a former ‘coloured group area’ in the main town of the Cape Winelands district adjacent to a provincial hospital. This community health centre serves the town and surrounding farming district. Unusually dedicated practice teams offer continuity of care, family-oriented care and the integration of chronic and acute patients. The practice teams depend on effective collaboration between the clinical nurse practitioners and doctors [19].

Sites D and E are clinics that lie just outside of a rural town in the Witzenberg sub-district. Agriculture, predominantly fruit farming, is the main local industry and consequently many of the study participants were farm workers. Site D is in a former ‘coloured group area’ and Site E in a former ‘black group area’. Both are under-serviced islands away from the hub, designed to supply labour for town residents and farmers.

### Formation of the co-operative inquiry group

The co-operative inquiry group consisted of five people, although participation varied according to availability. The first author engaged fully with the implementation of the protocol. Facilitation of the group process was primarily the responsibility of the second author, who did not engage with the implementation of the protocol. Four members were engaged with implementing the protocol. Members who implemented the protocol were chosen for being registered professional nurses with relevant language skills and interest in IPV. They are referred to below as the study nurses.

### The protocol for IPV management

Comprehensive assessment and management was performed according to the protocol [18]. Originally this had been conceptualised as a specialist service. It involved: universal screening for IPV; a supportive primary care provider relationship; systematic history of abuse and any attempts to enlist assistance from police, legal service providers or courts; forensic documentation; referral and reporting of abuse to the justice system; emotional status; participant’s verbal report of previous results of voluntary counselling and testing for HIV; casefinding for pregnancy and STIs; other special investigations as indicated; safety assessment; safety planning; referral to local services and follow-up appointment. All of this was conceptualised in the protocol as the responsibility of the primary care provider.

### Engagement with action by co-operative inquiry group members

Initial training of co-operative inquiry group members included an overview of: IPV; the study purpose; action research methods; use of the protocol; how to collect forensic evidence; the Domestic Violence Act of 1998; use of mental health assessment guides; and communication skills.

Primary care providers from the facilities were trained on site by the principal researcher to screen for IPV and refer to the study nurse. Providers were equipped with a laminated menu of possible screening questions and were requested to ask one of every adult female over 18 years [18].

Screening for study participants was performed over a period of four to eight weeks at each facility and 168 women were referred to a study nurse for assessment and management as per the protocol. Women were then invited to give feedback on their experience and 74 per cent returned for the follow-up visit one month later when they gave feedback to a different member of the inquiry group. On-site support and mentoring of the study nurses was provided by the first author, who implemented the protocol at one site and conducted follow-up interviews at another.

Five focus groups were conducted with the primary care providers to explore their experience with screening patients. These findings are reported elsewhere [20]. One focus group interview was held in the urban area with a male psychiatric nurse, four female clinical nurse practitioners, an emergency/trauma-room nurse and a female doctor. One rural focus group interview was held with doctors and nurses who had referred women to the study and another focus group interview with those who had not made any referrals. Both groups included a spectrum of doctors, from specialist family physicians to medical officers, and psychiatric, maternity and primary care nurses of both genders and all races. A final rural focus group interview included two lay counsellors, a nurse manager and a clinical nurse practitioner, all female. Interviews explored their experience of screening and initial management of identified women.

Sixteen key informant interviews were conducted. Participants were purposefully selected on the basis of their expertise. Eight interviews were held with members of the Department of Health who included the facility managers, relevant programme-specific managers and the head of chronic care for the Western Cape Province. Six interviews were held with academics working with gender issues or forensic medicine. Two interviews were held with leaders from non-governmental organisations with a specific focus on IPV [21]. These interviews explored different perspectives on how the emerging model for addressing IPV in primary care could work. Interviews were digitally recorded, transcribed verbatim and analysed using the framework method [22].

### Reflection by co-operative inquiry group members

Each co-researcher kept field notes to record key experiences, thoughts, emotions and reactions. Five co-operative inquiry group meetings were held over a period of 14 months for collective reflection and planning. All discussions were digitally recorded and transcribed. The first author circulated a summary after each meeting.

### Building the final consensus

The first author reviewed all transcripts and field notes and conducted a qualitative content analysis. A final meeting six months later provided an opportunity to reach consensus on the proposed model [21]. Thereafter a consensus of the group’s learning was circulated for validation.

### Ethical considerations

Ethical approval was obtained from the Health Research Ethics Committee of Stellenbosch University (reference no 6/10/216) and permission to conduct the study from the Department of Health. Where requested, a psychologist provided support to co-researchers experiencing vicarious traumatisation.

## Results

### Role of primary care providers

The protocol recommended that all women should be screened by primary care providers. In practice, however, primary care providers selected women for screening. It was clear that primary care providers did not support universal screening, but some were willing to consider the hypothesis of IPV when the patient’s presentation indicated it as a possibility:

*“How are we specifically going to identify that? It doesn’t fit in with any … we have enough to keep us busy the whole day. We don’t really have time to pay proper attention to it and to explore the problems further.”* (FGMW; 5:10–12)

*‘… that went way above my head. Ask every patient! I sort of probably decided on the first day I’m only using this for certain patients.”* (FGBLG; 16:16–18)

Most primary care providers were nurses with a task-orientated and bio-medical approach to assessing patients, who worked in clinics with high workloads. Primary care providers often felt they needed to protect themselves against further demands from patients or managers:

*“There is not a culture of support for health providers. They are ordered to do more and more tasks with no extra staff. Providers expressed feeling overwhelmed, exhausted, frustrated and unsupported … that spills out onto the patients where they’re not really that supportive of patients.”* (CIG 6, p.7, l.9-11)

*“… many times … providers do want to, but they know what is waiting outside the door … so we should not label all health workers as disinterested, its actually the system itself that squeezes them till a point that they can’t …”* (CIG 6, p.20, l.7-14)

An approach of selective case finding rather than universal screening, initial clinical care and referral to someone with the capacity to provide more comprehensive assessment and counselling appeared to be the best fit with the current realities of South African primary care.

*“If health workers know their role is to identify the issue and only provide clinical care before referring client to the intimate partner violence champion it actually fits in with their paradigm “I have identified this and can … move it over there!”* (CIG 6, p.16, l.39-42.)

*“The quality counselling we gave isn’t feasible to expect from others. I noticed psychiatric nurses don’t do it, social workers, nurses, and doctors also don’t!”* (CIG 6, p.18, l.1-4.)

Such feedback resulted in a modified protocol relying on a selective case finding approach based on cues identified from analysis of participants’ medical records. The identification of these cues from the medical record is described more fully elsewhere [20].

The original protocol suggested a number of different screening questions. However, a new question, “Are you unhappy in your relationship?” was thought to work best as it was “*harder for people to disagree with that statement if it is true for them.”* (CIG 6, p.26, l.10-13). “How are things going in your relationship?” was also considered an appropriate option.

### IPV champions

It was recognised that the inquiry group members who performed the assessment instinctively worked with a guiding style [23]. The full assessment took 60–90 minutes, which is impractical for primary care providers who are expected to spend an average of seven minutes per patient [24]. Nevertheless, women benefited from telling their stories to an empathic listener as well as from specific assistance:

*“It must be someone who wants to do the work; who is interested, who is motivated; who will give that listening heart and support …”* (CIG 6, p.18, l.11-13)

Reflecting on the attributes needed in a provider who attends to IPV, the inquiry group agreed that such a person could be any member of the multi-disciplinary team with the following characteristics: desire to work with IPV; empathy and good listening skills; respect for client confidentiality and autonomy; efficient case manager; collaborative approach to problem-solving; and effective multi-disciplinary team work.

Implementing this model of IPV care therefore would imply that such a person should be identified and set aside for this work in the Department of Health or Social Development, both of whom have some responsibility for the issue:

*“I think it is an excellent idea to use somebody outside because in the clinics it’s really hectic and I think this system (referring out) will be very much helpful for the client.”* (CIG 6, p.5, l.3-5.)

### Four key components

The inquiry group categorised the different components of the protocol into four broad areas: clinical, social, psychological and legal. The model that emerged was that the clinical component could be implemented by primary care providers, while the others required an IPV champion, as described above.

**Figure 1:**
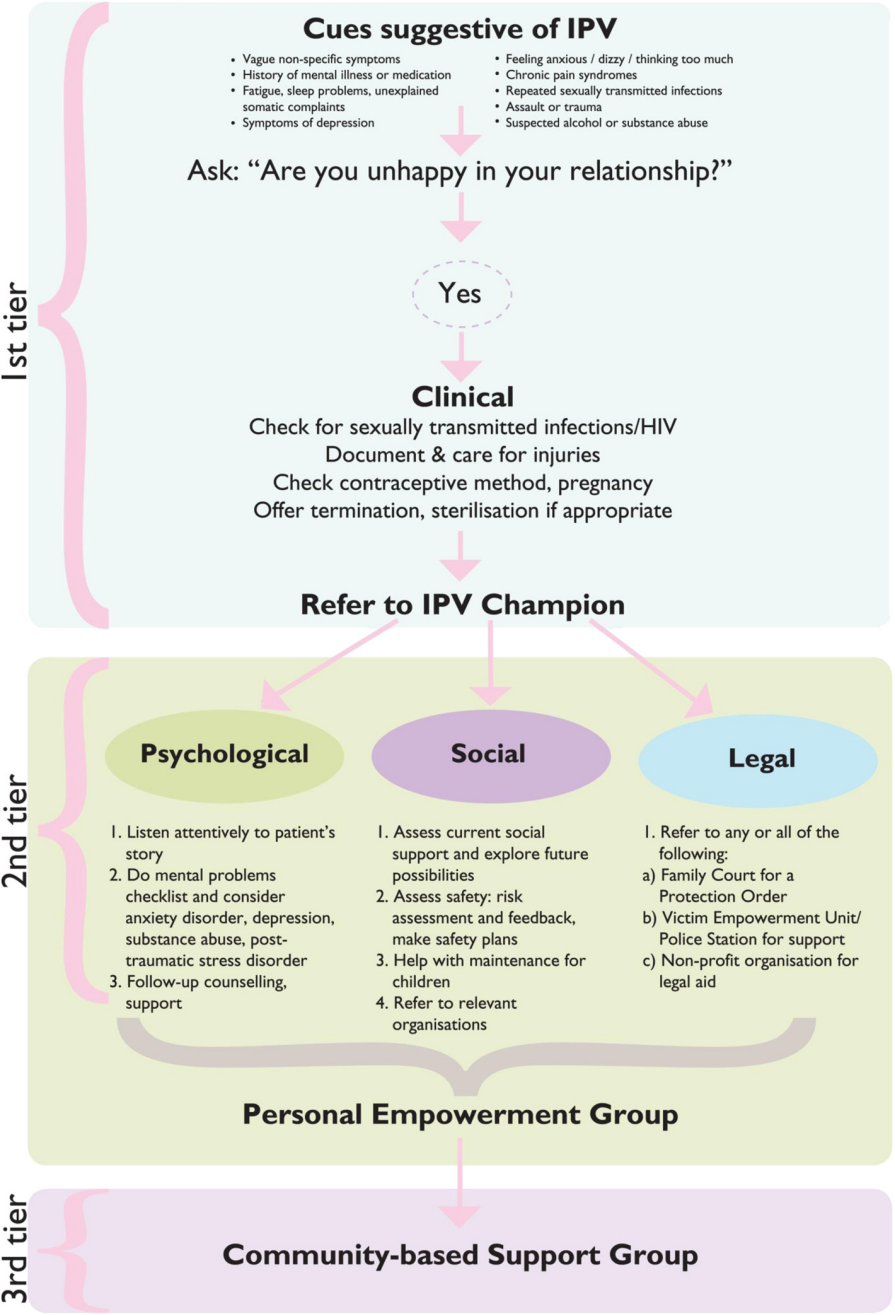
**Joyner’s Intimate Partner Violence Model.** Joyner’s Intimate Partner Violence Model. 1 page figure of model described in article.

The clinical component consisted of recognising cues suggestive of IPV as an underlying issue (Figure 1), treating injuries, forensic documentation if necessary, attention to unwanted pregnancies (including family planning, pregnancy testing or termination), syndromic management of STIs and HIV testing. This work is already an accepted part of the primary care providers’ role.

The social component comprised a detailed and empathic history of the abuse, an assessment of the woman’s immediate risk of injury or death, mobilisation of social support and planning for emergency situations. Survival issues such as procuring maintenance for children and disability grants were included.

The psychological component comprised screening for common mental health problems (depression, anxiety, post-traumatic stress disorder, substance abuse) and referral for more formal diagnosis and treatment if needed.

The legal component included a history of previous attempts to utilise the police or courts, information about legal rights and assistance to obtain a protection order or lay a charge. Referrals from study nurses significantly improved women’s chances of success with accessing legal rights. Victim empowerment units at police stations also played an important part.

### Assessment of abuse

After testing the protocol in the first phase of data collection, we reordered it to better fit our experience of the interview’s flow. The original protocol was forensically oriented. For example, abuse was framed as assault that would require forensic documentation using body charts. Yet only a third of the urban sample were injured, so we reformulated the forensic component as an appendix for ease of use, and tested it in the rural phase of data collection which followed [21].

The first phase of data collection also suggested the need for a more comprehensive assessment of the patient’s social context and so a genogram was inserted as part of the consultation to document the patient’s household and family context. See the Additional file 1 for our modified protocol.

### Danger assessment

Use of the safety assessment and plan in the original protocol exposed grounds for doubt about its validity in the South African context. It seems based on the assumption that the IPV survivor is badly injured, and cannot return home to an abuser, who may kill her. Our experience was at odds with this. Only two survivors in our sample moved to safe housing, for example, and many were not living with the perpetrator but needed to stop him coming to their home.

A quarter of perpetrators were at home reflecting South Africa’s high levels of unemployment. Consequently the accuracy of the scoring for danger assessment in this context seemed questionable since “Has he threatened to kill you?” scored the same as “Is he presently at home?” (see Additional file 1).

The scoring also emphasises the presence of a firearm. Ownership of a legal gun, combined with being a security worker, has been found to dramatically increase risk of femicide [25]. Yet paradoxically, while a firearm suggests a higher risk of injury or death, the absence of a firearm does not necessarily imply lesser risk.

The inquiry group members debated whether it helped a woman, who has nowhere else to go, to be told she is at severe risk. However, feedback from participants indicated that the safety assessment rating had helped them to reframe their situation from a different perspective.

As the assessment actually measures a woman’s risk of repeated assault or murder, the inquiry group felt that “*danger assessment*” would be a more accurate description of this process. The group felt that a woman’s risk of suicide, and even matricide, should be part of a danger assessment. In general the inquiry group felt that the validity of the items and their scoring to determine risk of injury or death needs further evaluation.

A revised danger assessment is found in the Additional file 1.

### Safety planning

The protocol’s suggestions for safety planning were based on relatively affluent circumstances. Access to credit or bank card and car keys did not match most participants’ socio-economic reality. However, participants reported that it had been helpful to learn about the importance of having one’s critical documents in order and copies hidden safely, as well as a bag packed.

The safety plan appeared to have more relevance when there was extreme physical violence. Also, given the danger abusers present when their partner leaves them, and for two years afterwards [26], key informants stressed the importance of a careful history and to inform her that if she is planning to leave him, she must tell no one.

### Need for better mental health assessment and care

At the outset the authors realised that the original protocol over-emphasised forensic issues and under-emphasised mental health care. Women survivors of IPV have a high prevalence of mental disorders [8]. The co-operative inquiry group therefore incorporated a more comprehensive component on mental health problems based on the World Health Organisation’s toolkit for the recognition, diagnosis and treatment of mental disorders in primary care. The second author had previously adapted this toolkit for the South African primary care context. This included a checklist to identify the possibility of a mental disorder [27]. Women were then referred to a mental health nurse or doctor to make a formal diagnosis and treatment plan. The decision to expand the section on mental health was validated by the experience of assessing women and reviewing their medical records:

*“The thing that jumps out at me is that psychiatric medication is number four on the list [of reasons for encounter]… That’s bizarre doesn’t happen in any other primary care grouping.”* (CIG 6; p.24, l.18-21)

Although the mental health nurse was the most obvious person to refer patients to, there were concerns with her/his capacity to deal with IPV:

*“I got the sense that the psychiatric nurse would value other things over something like domestic violence … they might put more of a premium on your regular psychiatric disturbances/conditions*” (CIG 6, p.5, l.15-18)

Alcohol abuse was the commonest substance women admitted to using. Given the prevalence of chronic pain in this sub-population, it was decided to add use of analgesia to the substances participants were questioned about [28].

### Need for ongoing containment and personal empowerment process

The inquiry group recognised that IPV is most often dealt with as an acute event in primary care, but is more applicable to a chronic care model. Women may take time to decide on what to do and may require support through multiple attempts at changing their lives. A once off comprehensive assessment and counselling session with referrals is unlikely to be sufficient.

The concept of IPV champions was congruent with emerging local chronic care policy which promoted ‘champions’ who provide continuity of leadership for chronic care and practice. In addition the local chronic care program, which mainly focused on non-communicable diseases, had produced a five-week personal empowerment process focused on issues of self-efficacy, self-care and motivation to change. In the final inquiry group members felt that this could help women with IPV and should be included in future interventions (see Additional file 2).

### Need for ongoing community-based support groups

Following such a personal empowerment process the inquiry group recognised the need for longer term social support. In fact many women spoke about their desire for a support group and even their willingness to establish one and be trained as a facilitator:

*“… something valuable about a group process, especially for isolated, depressed, marginalised women. There’s a lot that can really help them to value themselves more, to feel more connected, to be supportive to each other.”* (CIG 6, p.11, l.14-24)

A system of support groups can be beneficial in scarcely resourced contexts with overburdened health systems. There is also scope to incorporate job skills development as a lack thereof entraps many women. Adolescents were reported to “*fall through the gaps*” within contemporary health systems where their needs are very poorly met. Further, the onset of IPV difficulties in teenagers was emphasised by key informants [21]. Given the stigma that surrounds IPV, the group could be referred to as a women’s health or interest group. Support groups were not implemented during the study period.

## Discussion

### Key findings

The model of care for women with IPV that emerged from the co-operative inquiry group process is illustrated in Figure 1. The first tier of the approach relies on the primary care provider, usually a nurse, to recognise cues suggestive of IPV. An analysis of the women’s medical records and review of the literature suggested the cues shown in Figure 1[20]. When these cues are recognised the provider asks a question such as “Are you unhappy in your relationship?” If IPV is disclosed as an underlying issue then the provider will deliver important clinical aspects of care and refer to a local IPV champion. We developed a one page flow chart for assessing and managing such patients to prompt the provider in the hope of moving from the fragmented approach of the past to a more clearly defined package of clinical care.

The second tier of the approach relies on an IPV champion. Two IPV champions (as back up and support for one another) may provide a service at the primary care facilities on a rotational basis throughout a sub-district. Here patients are assessed and assisted comprehensively and then invited to participate in a five week empowerment group. The IPV champion could be either someone set aside by the Department of Health, with the recommended attributes and motivation, or a social worker from the Department of Social Development who would work in collaboration with primary care services. Whoever is identified in a specific sub-district would provide comprehensive assessment and assistance by taking a history of abuse and attending to social, psychological and legal issues. Training of the IPV champion should pay equal attention to the therapeutic and motivational interviewing skills as well as expertise in the different areas of IPV management. The IPV champion should also facilitate the five-week personal empowerment group process following the initial assessment. A skilled facilitator in such a program could create a supportive environment for discussion of difficult issues and build awareness around protection orders, human rights, effective parenting, conflict management skills and so forth. It could offer a potentially powerful process of personal transformation, blending educational and therapeutic value to provide a context where women could be stabilised, educated and treated. Broader than the medical model, this group process could offer participants a rare opportunity to attend to the meaning of their own life, reflect on their behaviour, choices and way forward. Note, this program was not implemented by the inquiry group during the study period.

The third tier of the approach relies on the establishment of community-based support groups that would support women in the longer term. These groups could be established with the help of the IPV champions and the Department of Social Development as well as local non-profit organisations. Adolescents and young adults should have a group of their own, with women older than 25 years comprising another group. Both groups should initially be facilitated by a social worker, psychiatric nurse, occupational therapist or psychologist. Over time they may become self-sustaining.

### Comparison to literature

Four factors have been identified that increase provider self-efficacy for IPV screening: institutional support; effective screening protocols; thorough initial and ongoing training; and immediate access/referral to onsite and/or offsite support services [29]. Our model allows for easy access to onsite support via the IPV champion and recommends a constructive approach to effective screening. Previous reviews have only considered universal screening as a valid approach [29] and studies have been critiqued for focusing too much on whether programmes work and less on how they work [30-33]. Given the cues presented by women experiencing IPV and our experience of trying to implement universal screening in the South African primary care context, we argue that selective case finding of women with a higher risk of IPV is a more constructive approach in resource poor settings [20]. Clearly future implementation of the model will require institutional, and at best intersectoral, support as well as initial and ongoing training. Successful interventions, which alter provider behaviour, should address both predisposing factors (training of providers) as well as enabling factors (written protocols and prompts for the clinical context) [29].

When compared with Colombini’s typology of IPV models, the model suggested here is primarily one of “facility-level integration” in that a patient will receive initial screening, assessment, management and a small group empowerment process from different providers at the same facility [34]. Provider-level integration of care, in which a single provider offers a comprehensive service, is more typical of high resource settings and most of the literature refers to this model. For example individually tailored counselling sessions for pregnant women [35]; one-to-one advocacy interventions [36]; individual cognitive-based therapy [37]; and one-to-one advocacy for pregnant women [38]. Systems-level integration suggests a model where the service is offered in multiple sites within a system such as a sub-district. In the model suggested here the community-based support group component extends the service to the system level.

The communication style suggested in the model is typical of a guiding style – collaborative, empathic, evocative, supportive of choice and control and yet directive in its focus and process [23]. The guiding style is seen as appropriate when engaging with brief behavior change counselling. As these women survivors of IPV are facing difficult decisions about change and behaviour it is not surprising that this approach makes sense. One can therefore make a conceptual link between communication skills for consulting the IPV survivor, brief behaviour change counselling and motivational interviewing. This may be useful in the approach to training IPV champions. The structure of the 5As (ask, alert, assess, assist and arrange) that is currently seen as best practice for brief behaviour change counselling may be useful in the approach to discussing mental health problems, safety planning and use of legal rights and resources [39,40].

### Limitations of the study design

Mash and Buskens suggest quality criteria for action research [17]. In retrospect we were still too contaminated by an empirical-analytical mindset to fully innovate, experiment and implement change as part of the process. This dynamic was compounded by the fact that membership of the co-operative inquiry group was not consistent. Further, the group ownership of the inquiry process had to be held in tension with the first author’s requirements for a doctoral study. The development of reflectivity also varied between co-researchers.

### Implications and recommendations

The intervention depicted in Figure 1 is currently being piloted in the rural Witzenberg sub-district of the Western Cape as a collaborative project between the Departments of Health and Social Development. The piloting of this IPV model will involve further monitoring and evaluation and will be reported on. If the model is successfully implemented then the Department of Health intends to implement more widely.

Further work should also be undertaken to look at the incorporation of this approach to IPV into the HIV and TB programmes, which are often separate vertical programmes in South African primary care. The incorporation of the model into emergency and perinatal units within community health centres should also be considered.

## Conclusion

This study demonstrated the feasibility of implementing a model for recognising, assessing and assisting women survivors of IPV in South African primary health care. The original protocol had to be adapted in terms of its expectations of the primary care providers, overly forensic orientation, lack of depth in terms of mental health, validity of the danger assessment and safety planning process, and need for ongoing empowerment and support. The inquiry process led to a model of comprehensive and intersectoral care that is integrated at the facility level and which is now being piloted in the Western Cape, South Africa.

## Abbreviations

IPV: Intimate Partner Violence; HIV: Human Immunodeficiency Virus; AIDS: Acquired Immune Deficiency Syndrome; STI: Sexually Transmitted Infection; TB: Tuberculosis.

## Competing interests

The authors declare that there are no competing interests.

## Authors’ contributions

Conception and design: KJ and RM. Inquiry group process: KJ, RM and co-researchers. Further analysis of data: KJ. Interpretation of data: KJ and RM. Final approval of the article: KJ and RM. Both authors read and approved the final manuscript.

## Authors’ information

Dr Kate Joyner’s voluntary work during the States of Emergency in counselling, training and public education for Rape Crisis and helping to establish the first crisis clinic for political detainees (1987) and feminist shelter for battered women (1985/6) in Cape Town, has matured into a lifelong commitment to enable the provision of sensitive, compassionate care to those affected by violence in our society.

Prof Bob Mash is Head of Family Medicine and Primary Care at Stellenbosch University and is supporting Dr Kate Joyner in her work on IPV. He has expertise in action-research and co-operative inquiry.

## Pre-publication history

The pre-publication history for this paper can be accessed here:

http://www.biomedcentral.com/1472-6963/12/399/prepub

## Supplementary Material

Additional file 1 **Modified Protocol.** IPV assessment tool for use in 1^st^ stage of 2^nd^ tier of model. Click here for file

Additional file 2 **Personal Empowerment Group.** Personal Empowerment Group Programme. Click here for file
