# Supplementary material for: A comprehensive model for intimate partner violence in South African primary care: action research
Source: BMC Health Serv Res. 2012 Nov 14;12:399. doi: 10.1186/1472-6963-12-399 (PMC3534545; doi:10.1186/1472-6963-12-399)
Supplement: Additional file 1 — Modified Protocol. IPV assessment tool for use in 1st stage of 2nd tier of model. [file 1472-6963-12-399-S1.docx]

## Appendix 1

## Folder No: ______________ Date: _________________

### CONFIDENTIAL REPORT ON IPV EXAMINATION

**PATIENT INFORMATION:**

Name: ______________________________ Age: _____________

Current Residential Address: __________________________________________

Tel No: (h): _____________ (w): _____________ (cell): ____________

Safe to phone? ____________ _____________ ____________

Partnership Status: Married how? ___________________________________

Same-sex partner ⬜ Single ⬜ Co-habiting ⬜ Divorced ⬜

Genogram:

#### HISTORY OF MOST RECENT ABUSE

Location, date and time of incident:

______________________________________________________________________

Identity of abuser: _________________________________________

Nature of the abuse

**PHYSICAL EMOTIONAL SEXUAL FINANCIAL**

Hitting ⬜ Being insulted ⬜ Unwanted Touching ⬜ Withholding Money ⬜

Kicking ⬜ Shouting ⬜ Infidelity ⬜ Taking Money ⬜

Use of a Restricting contact Sexually transmitted Controlling All

Weapon ⬜ with Family/Friends ⬜ infections ⬜ Financial Decisions⬜

Pushing ⬜ Threats ⬜ Forced Intercourse ⬜ Other _____________

Choking ⬜ Controlling her Other _________________________________

Activities ⬜

Burns ⬜ Other ___________________________________________________

Other______________________________________________________________________

Patient’s description of most recent abuse (Use exact words as far as possible. Describe severity of abuse.)

**Other episodes of abuse:**

Describe frequency & severity of past abuse, using direct quotes from the patient. Describe mechanism, location and extent of injury and/ or symptoms/conditions.

Frequency over last 2 years: 0-10 / 11-20 / >20

Patient’s description: ___________________________________________________________________________

___________________________________________________________________________

___________________________________________________________________________

___________________________________________________________________________

___________________________________________________________________________

___________________________________________________________________________

#### IF RELEVANT:

Has a criminal charge been laid? Yes No

If yes, what charge was laid?

Assault ⬜ Grievous bodily harm ⬜ Rape ⬜ Indecent assault ⬜

Contravention of protection order ⬜

Name of police station:_______________________ CAS No: ______________________

If no, does the patient intend laying a charge?

Yes No Unsure

Does patient have a Protection Order? Yes ⬜ / No ⬜

Name of magistrate court:

If no, intends to apply for protection order; Yes ⬜ / No ⬜ / Unsure ⬜

**ASSESSMENT OF EMOTIONAL STATUS:**

#### Mental Problems Checklist

1. Are you thinking too much? ………………………………………...................... ⬜
2. How are you sleeping at the moment? ………………………………................... ⬜
3. Do you feel exhausted or tired even when you are not working hard? …… ........ ⬜
4. Do you feel sad or like crying for no reason?…………………………................ ⬜
5. As a person there are things that you enjoy doing – do you find that you no longer

enjoy these things? i.e. listening to music or going out with friends…..…........... ⬜

1. Do you sometimes have the feeling as though you are going to hear bad news?… ⬜
2. a) Have you ever felt you should cut down on your drinking? ..………………. ⬜

b) Have people annoyed you by criticising your drinking? ………………….. ⬜

c) Have you ever felt bad or guilty about your drinking?……………………… ⬜

1. Have you ever had an eye-opener first thing in the morning to steady your

nerves or to get rid of a hangover?…………………………………………… ⬜

1. Have you experienced traumatic events that made you feel extremely threatened or

endangered? Or witnessed someone else in this situation?…………………….. ⬜

1. Have you used substances such as dagga or tic? Any others? (e.g. mandrax, glue, benzene or solvents, ecstasy, cocaine/crack)

In the last month? ............. In the last year? ....................................................

**10.** How many days last month did you take painkillers?........................................

**If positive to any one further assessment may be required.**

If positive to **2,3,4,5** consider **depressive disorders.**

If positive to **1, 2, 6** consider **anxiety disorders.**

If positive to **7** consider **alcohol use disorders.**

If positive to **8** consider **post traumatic stress disorder.**

If positive to **9 or 10** consider **substance abuse disorder.**

**REFERRAL FOR MENTAL HEALTH**

Depression requiring further assessment? Yes No

Post traumatic stress disorder requiring further assessment? Yes No

Anxiety disorder requiring further assessment? Yes No

Alcohol/substance use disorder requiring further assessment? Yes No

**DANGER ASSESSMENT:**

A danger assessment must be done for all patients who disclose domestic violence. Record answers to the following questions. The answers to the questions can be assigned a value for risk assessment. Assign a value for the answers as follows: No = 0 Yes = 1

## Add up the total to provide a risk rating: 1 – 2 🡺 Caution

## 3 - 5 🡺 High Risk

**6 -11 🡺 Severe Risk**

Has he threatened you with physical violence? Yes No

Has he threatened the children with physical violence? Yes No

Is there a firearm in the house? Yes No

Has he threatened to kill you? Yes No

Has he threatened to kill the children? Yes No

Does the patient think he is capable of killing her? Yes No

Were alcohol and / or drugs consumed prior to the last incident of abuse? Yes No

Has the abuse escalated in either frequency or severity? Yes No

Have you ever received medical treatment for injuries sustained

as a result of abuse? Yes No

Have you ever thought of killing yourself? Yes No

Have you ever thought of killing the children? Yes No

Score: 1-2 3-5 6-11 **TOTAL RATING**

**SAFETY PLAN:**

Help the client think and plan the following:

**?** Where can you go if you need to leave home?

**?** Who can you trust to tell about the domestic violence?

**?** Where can you leave money, clothing, copies of documents and valuables if necessary?

**?** How will you ensure your children’s safety?

Advise patient to hide a readily available packed bag with essential items such as:

- Her ID book, birth certificates, hospital cards, and other important documents.
- Keys, money and bank card.
- Clothes for her and her children.
- Important telephone numbers.
- Copy of the protection order and suspended warrant of arrest if she already same.

Make a list together of other things to take with her, for example:

- Children’s favourite toys, books.
- Children’s school books and uniforms.
- Toiletries
- Other valuable personal items, for example, photos and jewellery.

##### REFERRAL LEGAL AND PSYCHO-SOCIAL ISSUES

Referral letters supplied? Yes No

Magistrate court for protection order Yes No

Police station for criminal charge Yes No

Counselling Yes No

Legal support Yes No

Social worker Yes No

Shelter Yes No

Other:______________________________________________________________________

**FOLLOW UP:**

Date and time of appointment if made for follow- up? _________________________________

Signature: ________________________________

Health Facility: ________________________________

Date: ________________________________
