# Supplementary material for: A comprehensive model for intimate partner violence in South African primary care: action research
Source: BMC Health Serv Res. 2012 Nov 14;12:399. doi: 10.1186/1472-6963-12-399 (PMC3534545; doi:10.1186/1472-6963-12-399)
Supplement: Additional file 2 — Personal Empowerment Group. Personal Empowerment Group Programme. [file 1472-6963-12-399-S2.pdf]

Western Cape
Government

Health

# Lifestyle Intervention For Empowerment Programme

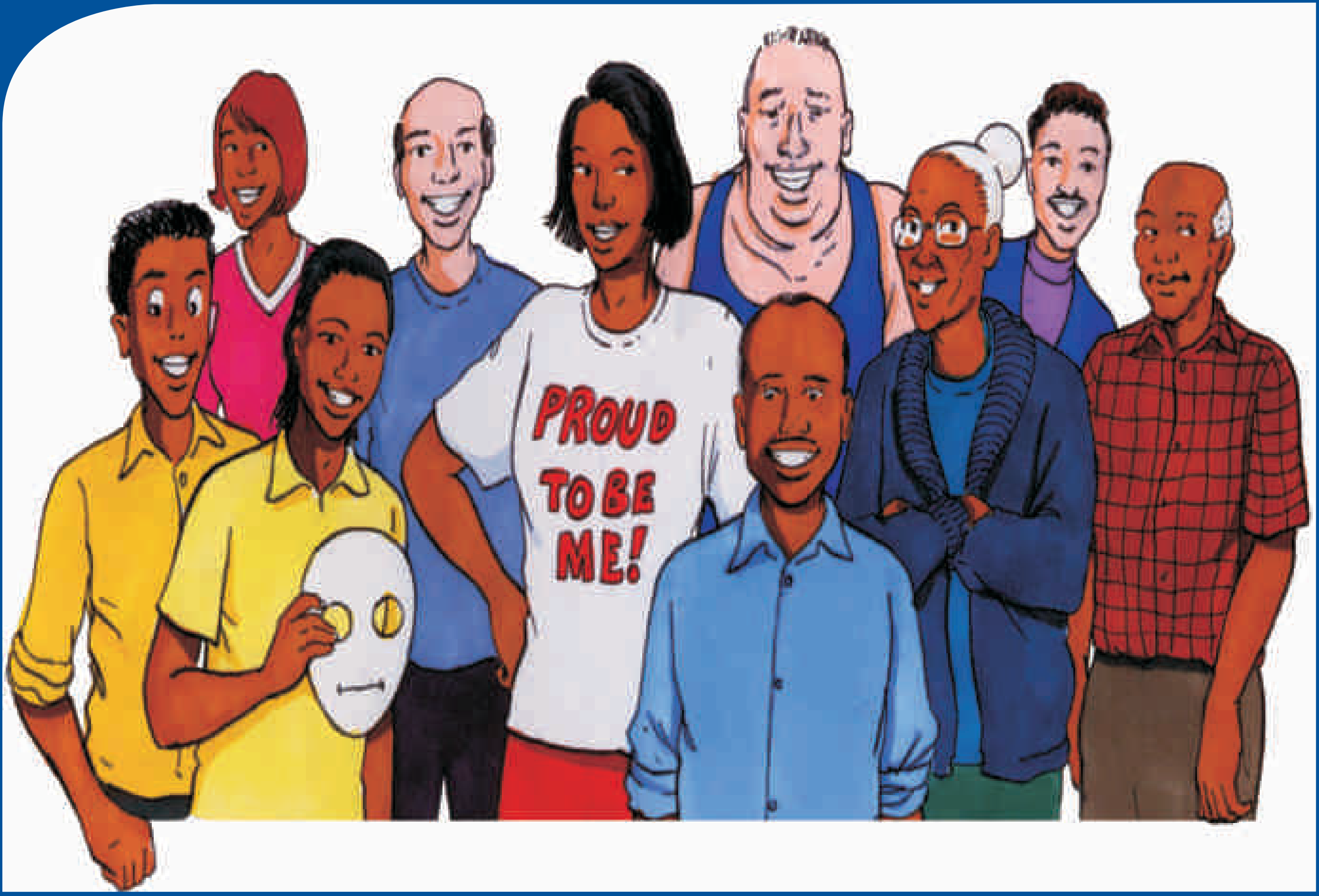

# PART 1

**AIM:** To change the biomedical paradigm of our primary health services from the biomedical model to a socio-ecological model. We will do this by empowering our clients to take control over their own health by changing their lifestyles, resulting in healing for both the clients and the greater community.

**Using the flipchart:** This flipchart comprises 5 modules:
Module 1: Posters 1a, 1b, 1c
Module 2: Poster 2
Module 3: Posters 3a, 3b, 3c, 3d, 3e, 3f
Module 4: Posters 4a, 4b, 4c, 4d
Module 5: Posters 5a, 5b, 5c, 5d
Module 6: No posters because this is a feedback session where the clients share their experiences.

Each meeting will comprise of a short presentation followed by a small group discussion of the presentation led by a facilitator. The small group discussion is usually more important than the presentation. Each meeting can also have an exercise session based on the capabilities of the clients. When using the flip chart remember that a picture is worth a thousand words. You can create your own stories around the pictures to illustrate the principles involved. The posters with words are put there more for you than for the clients. They are there to remind you to deal with certain issues. Everyone will present a flip chart differently according to their personality. There is no one right way of doing it.

**DISCUSSION:** This flipchart is designed to bring about behavioural change in our clients. Behavioural change is seldom achieved by telling people what to do. Rather, we are presenting a principle or concept to our clients and then asking them what they are going to do about it. After all, they are adults, and only they can take responsibility for their health. We are making the assumption that no one is stupid (although all of us do stupid things at times) and that we can trust our clients to make the decisions that are right for them. In essence, we are handing the power back to our clients. As health professionals we should not take the 'expert' role unless asked, but rather should focus on becoming agents of transformation in facilitating the empowerment of our clients, so that they feel in control, so that they participate in decisions and that they mobilise the resources needed to regain their health.

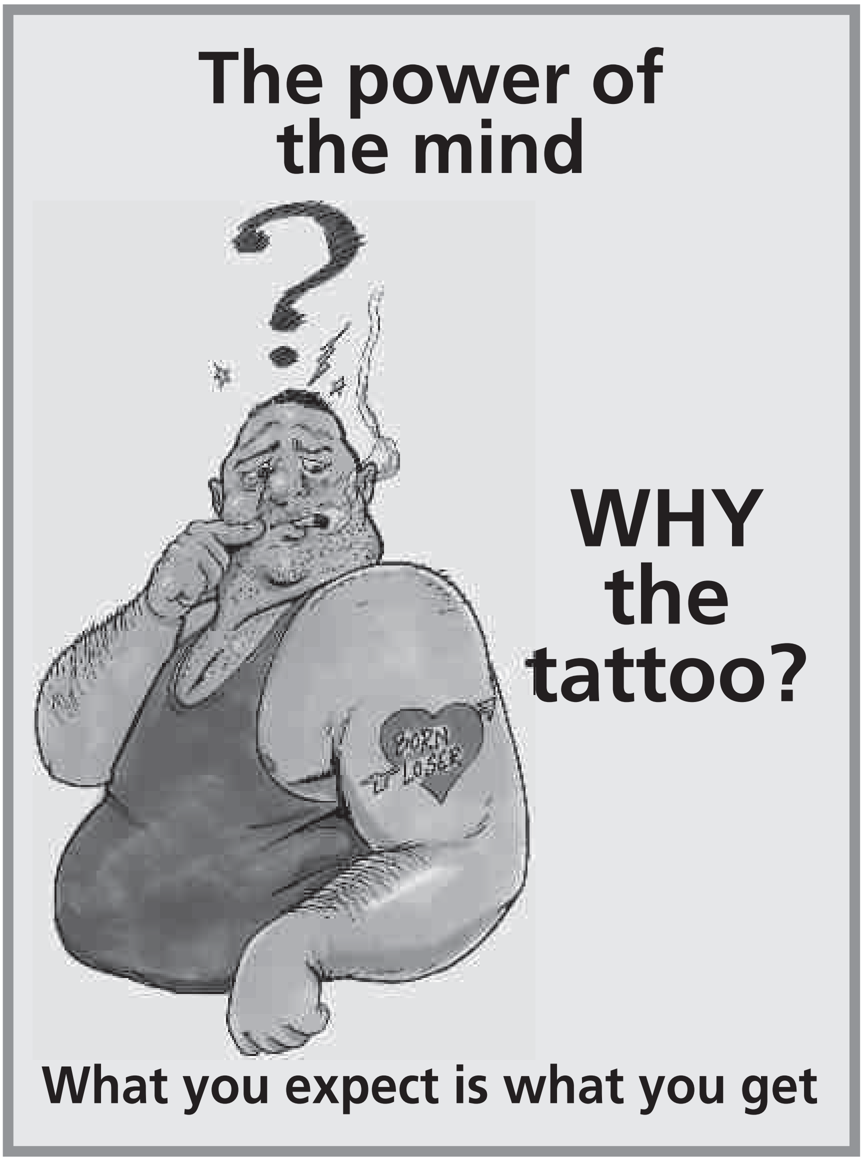


1a

**AIM:** To get our clients to understand the power of the mind

**STRATEGY:** Use a story e.g. a man is shopping in Hong Kong when he walks past a tattoo shop. In the display window he sees a tattoo with the words, “Born Loser". Intrigued as to why anyone in their right mind would ever be tattooed with something so depressing he enters the shop to enquire. Behind the counter is an old Chinese gentleman. He asks him about the tattoo. The old Chinese man replies, gesticulating with his index finger towards his temple: “Understand, before tattoo on arm, tattoo already in mind”. Feel free to change the details of the story if you so feel. Alternatively you can ask the clients why someone would have such a depressing tattoo on their arm.

**RATIONALE:** What you truly believe in and expect in life, is what you end up getting. We are using this principle in the context of setting goals, which is the next poster. It can also be used powerfully in the context of health seeking behaviour.

# The power of the mind

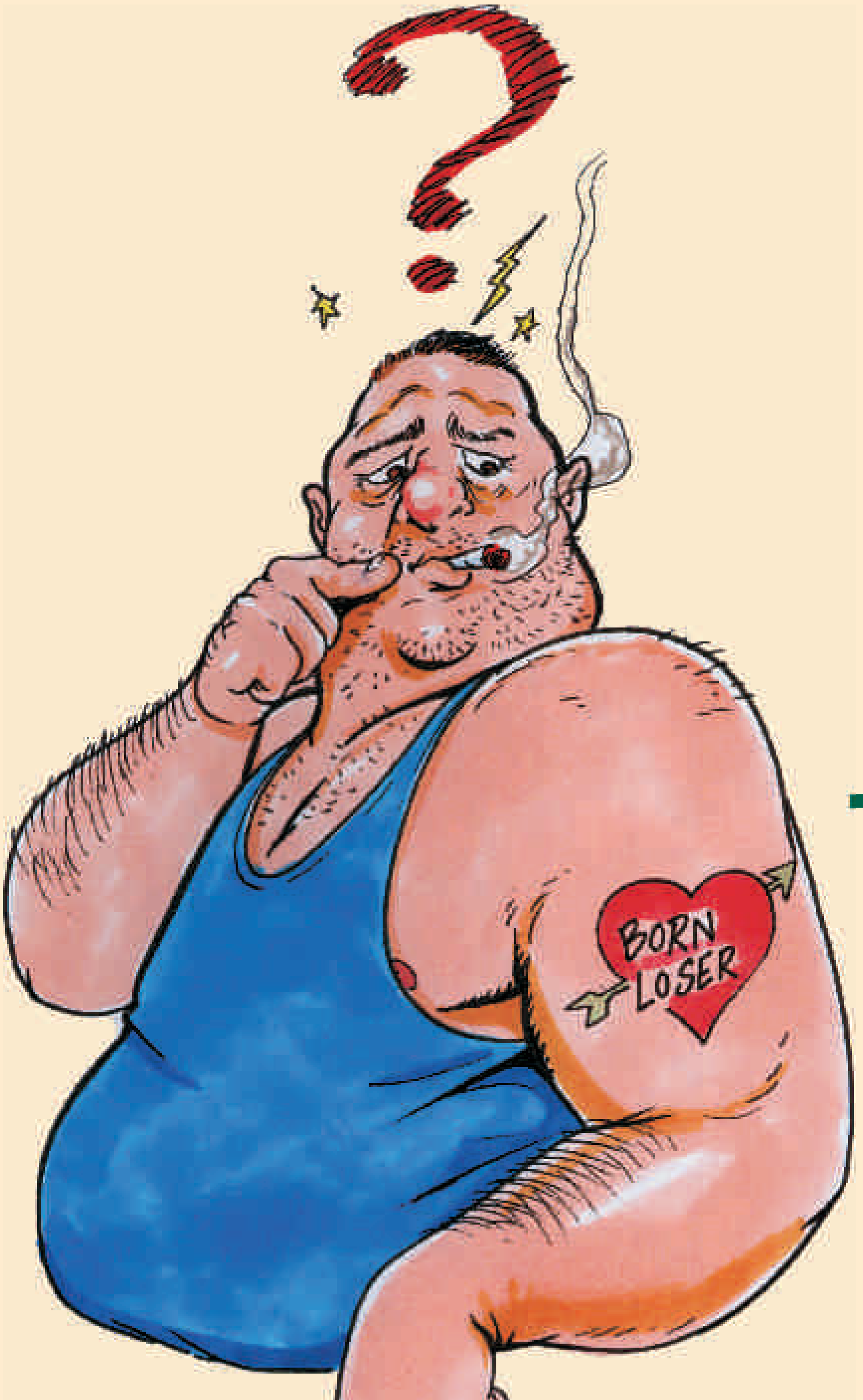


## WHY the tattoo?

**What you expect is what you get**

## Setting goals

- * What do you expect to achieve for → yourself / → others
- * See yourself as having achieved it
- * Work hard - it is then impossible to fail
- * Let your God be your partner

Write down your goals

1b

**AIM:** To get our clients to set a goal in terms of what they expect to get out of the programme. This should preferably be done in writing.

**STRATEGY:**

1. **What do you expect to achieve, for yourself and for others.**
   This is probably best dealt with in question form to the group, eliciting a variety of different answers so as to give people options. Each person will have their own individual goal, but if possible try and link all the goals in terms of an improved quality of life. An important point to make is that a selfish goal is limiting in the long term. We are far more likely to develop passion and good feelings around a goal if it benefits not just ourselves, but also our family or greater community.

2. **See yourself enjoying the benefits of having achieved your goal.**
   This can be done as a simple visualisation exercise. Get everyone to close their eyes and relax. Then get them to see themselves enjoying the benefits of achieving their goal e.g. sleeping better, less pain, able to climb the stairs without effort etc. This is a follow on from the previous poster, remembering that what you expect is what you get.

3. **See yourself as working hard at achieving your goal and realise that as you do so, it is then impossible to fail.**
   This can be done as a continuation of the visualisation exercise.

4. **Let your God be your partner.** This is part of the visualisation exercise.

   Points 2 - 4 (the visualisation exercise) can be done after the presentation is completed and clients have had time to write down their goals.

**RATIONALE:** Goal setting is one of the cornerstones in achieving success.

# Setting goals

* What do you expect to achieve for → yourself
  → others

* See yourself as having achieved it

* Work hard - it is then impossible to fail

* Let your God be your partner

**Write down your goals**

# How do you move a mountain?

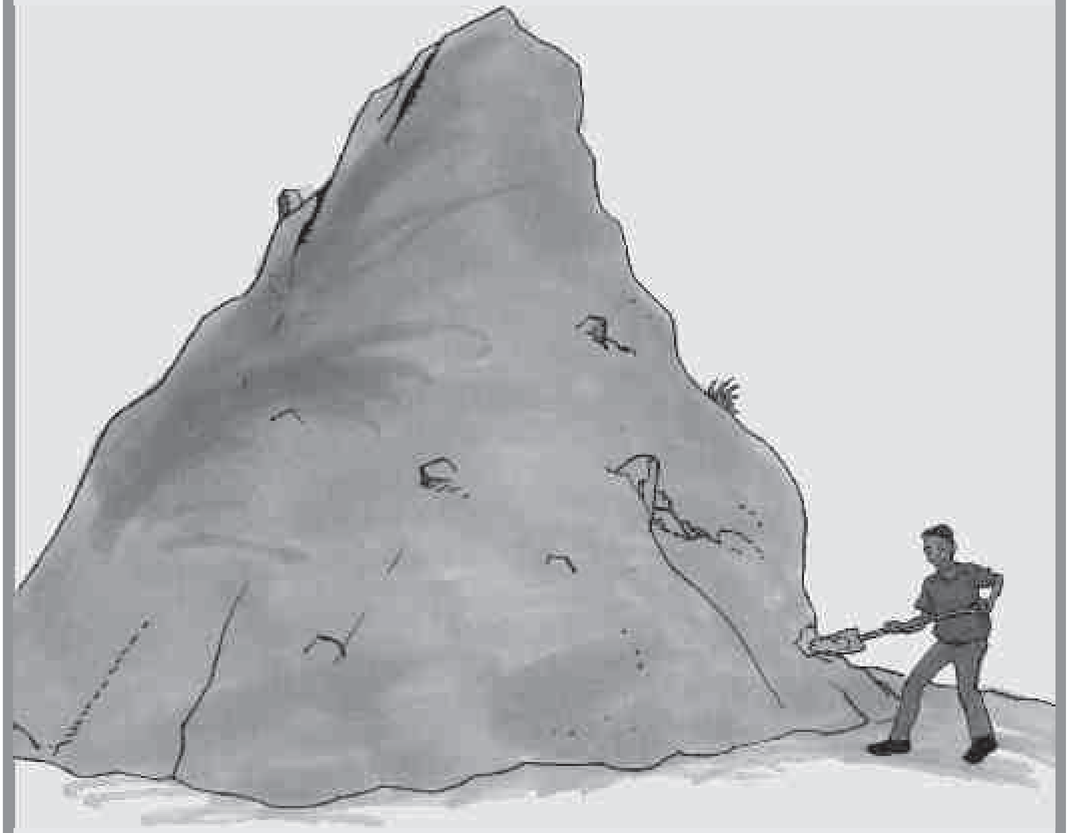

**Change is a process**

1c

**AIM:** To reassure our clients that they do not have to make any drastic changes, but that true behavioural change is a process.

**STRATEGY:**

**Q:** How do move a mountain?

**A:** One shovel full at a time.
Reassure the clients that they do not have to make any drastic changes. For instance, we do not want to see our clients going onto crash diets.

**RATIONALE:** Behavioural change is difficult and can usually only be successful in the long term if it is a process.

# How do you move a mountain?

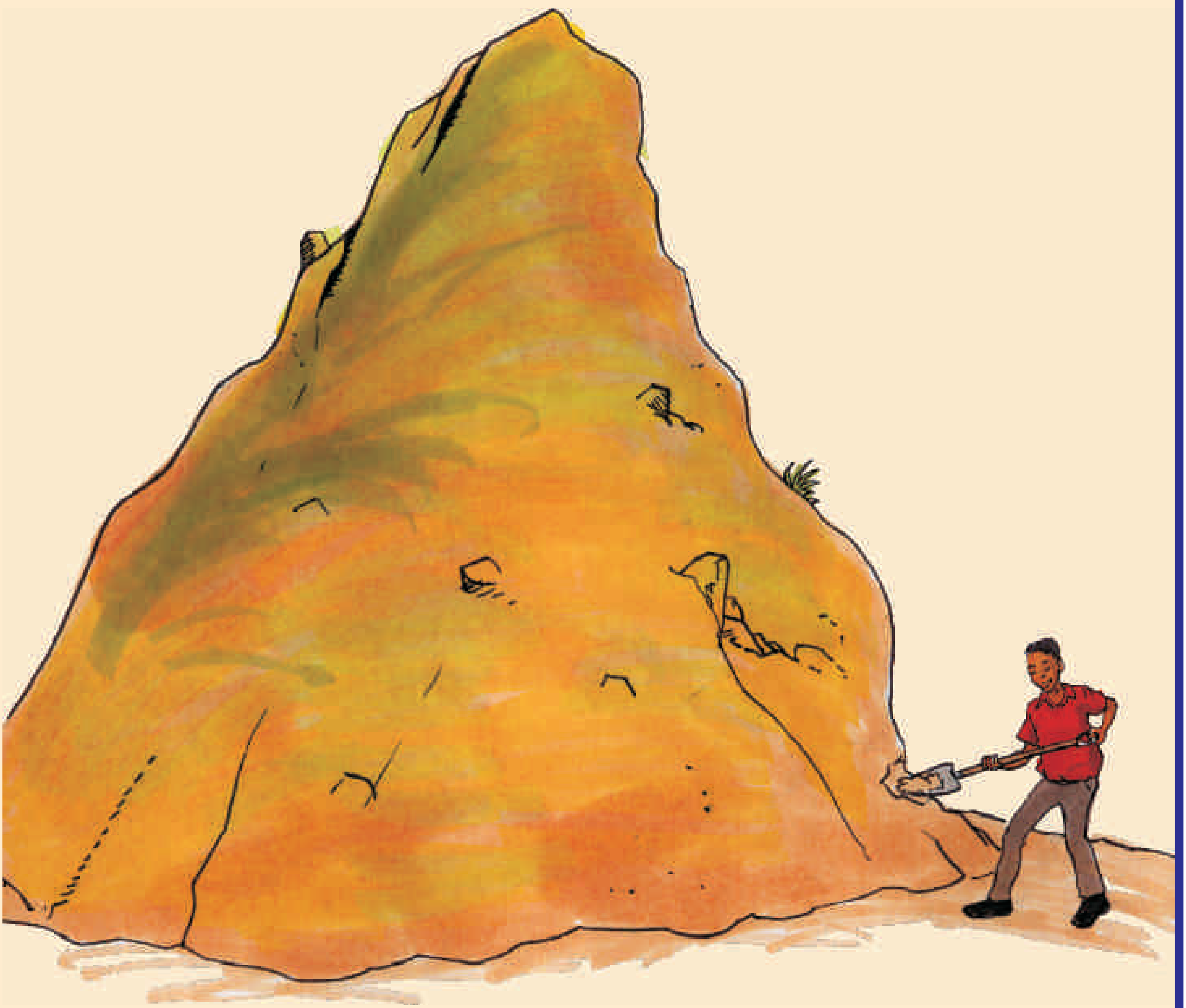

**Change is a process**

**Learn to live in the moment**

**What helps you to relax your body?**

**What helps you to relax your mind?**

**How do you calm your spirit?**

**Prescription:**
**Do the exercises**
**10 minutes twice a day**

2

# PART 2

**AIM:** To teach simple relaxation and meditation techniques

**STRATEGY:**

**Relax the Body:** Tense and relax muscles. Lie down, start tensing and relaxing the different muscle groups, beginning with the toes and ending with the scalp.

Teach abdominal breathing. The best way to start is to breathe strongly out through the mouth using the abdominal muscles to push in, then breathe in through the nose and allow the abdominal muscles to push out. Slow the breathing down otherwise the clients will start feeling light headed.

**Relax the Mind:** Learn to live in the moment by focussing on the breathing (slow it down). Focus also on smells, sounds, the feel of clothing on the skin etc. You are now meditating

**Calm your spirit:** Focus on something uplifting
Learn to breathe in good thoughts and breathe out bad thoughts. Focus on being thankful for the things you do have. A grateful attitude is one of the keys to a contented life.

**Tip:** Ask clients to breathe into the heart area.

**Prescription:** Do 10 minutes meditation twice a day.

**RATIONALE:** De-stressing is one of the keys to health.

# Learn to live in the moment

## What helps you to relax your body?

## What helps you to relax your mind?

## How do you calm your spirit?

**Prescription:**
**Do the exercises**
**10 minutes twice a day**

## Are you a victim?

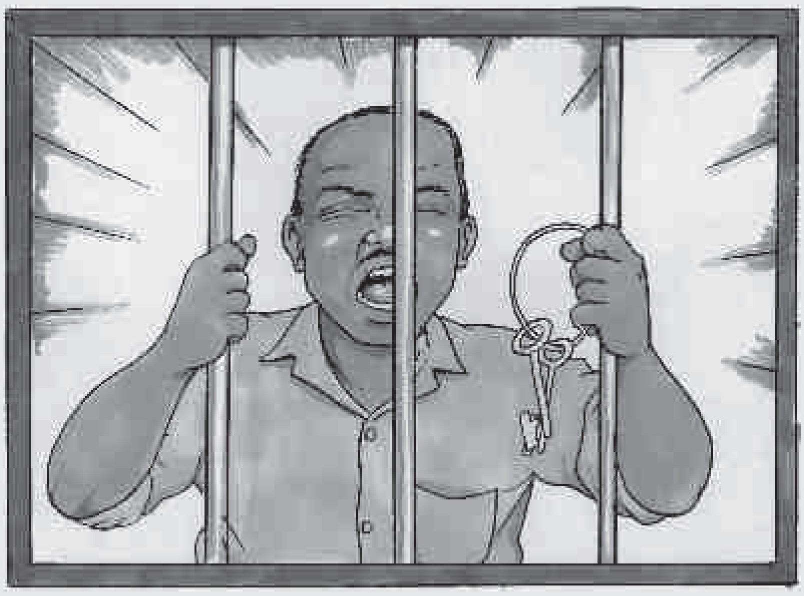

**To get out of jail, you need to know that you hold the keys**

3a

# PART 3

**AIM:** To challenge any of our clients who might hold onto a 'victim' mentality.

**STRATEGY:** Use the picture and ask the clients if they are victims. Many of us are in prisons of our own making. We do not realise that we hold the keys to get out of prison. Discuss with the clients how many people in the community play 'victim'. They blame everyone else for the trouble they are in. If someone else is to blame, then only the other person can sort out their problem. They do not realise that they have handed over their power to change to someone else by playing this game.

**To get out of jail you need to know that you hold the keys** - this leads us to the next poster.

**RATIONALE:** It is difficult to help someone with a 'victim' mentality. Change only comes when someone takes control of their own life.

# Are you a victim?

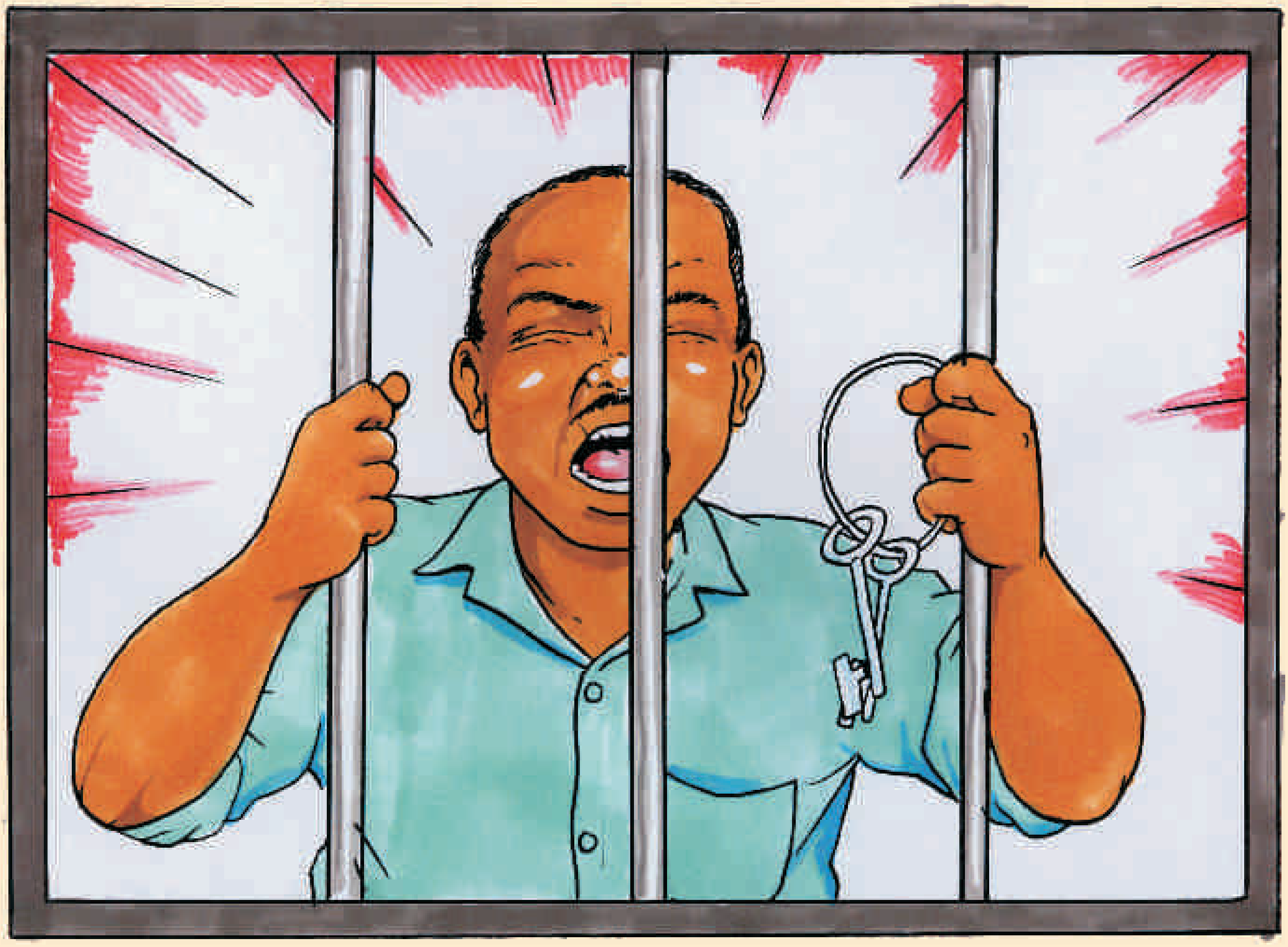

**To get out of jail, you need to know that you hold the keys**

**AIM:** To get clients to understand their basic psychological make up and understand how problems could arise in their lives if their basic psychological needs are not met.

> **We are all the same inside**
>
> **We all want to be liked and accepted by others**
>
> **We all want to feel that we are of significance, that there is meaning to our lives**
>
> **If these needs are not met, problems with health will arise**
>
> **3b**

**STRATEGY:** You can use the points on the poster, or you can take attention onto yourself by stepping in front of the poster and explaining the points. The poster in this case is there to remind you about the points you need to cover.

**We are all the same inside.** This is a key statement as this creates a safe atmosphere and no one feels we are picking on them. A good way to present these statements is to make the initial statement that we are all the same, that we all have problems and no one is better than anyone else, we just have different problems. Then ask the clients if this is true and elicit responses.

**We all want to be liked and accepted by others.** We all have inside of ourselves a 'little child'. This 'child' desperately wants to be loved and accepted by others. When our inner 'child' feels rejected or abandoned, we might do a lot of things we might not necessarily agree with so that we can be accepted by others e.g. taking drugs because all your friends are taking drugs, or being stuck in an abusive relationship because breaking up would be like a form of rejection. Only when this 'child' feels loved and nurtured can we begin to find contentedness in life.

**We all want to feel that we are of significance, that there is meaning to our lives.** A life without meaning is often a life without hope. Without a good reason to live, we all end up doing things that make us feel good in the short term, but might be bad for us in the long term e.g. if we are not saving towards something special, then it is easy for us to spoil ourselves by buying a lot of nice things at the beginning of the month and then struggle to meet the budget at the end of the month. If the things we do have meaning then we do them with enthusiasm, even if all we are doing is bending paper clips.

**If these needs are not met then problems with health will arise.** Because these are such deep psychological needs, if left unmet they will lead to problems with emotional or physical health in the long term.

**RATIONALE:** You cannot sort out a problem by dealing with its symptoms, you have to understand the real root of the problem and deal with that.

# We are all the same inside

**We all want to be liked and accepted by others**

**We all want to feel that we are of significance, that there is meaning to our lives**

**If these needs are not met, problems with health will arise**

**AIM:** To help our clients create an understanding of life which will help them to cope with life's problems.

## Giving meaning to life

**Where do I come from?**

**What am I doing here?**

**Where am I going?**

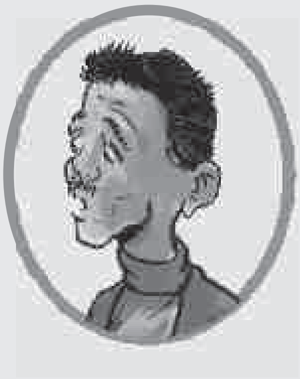

**Listen and follow what your heart is telling you**

**3c**

**STRATEGY:**

**How to give meaning to your life.**
Start by discussing some of the main philosophical questions that all of us ask at one stage or another.

**Where do I come from?** If your answer is that you were created by your God, then it implies that you cannot be rejected as he created you, he will never abandon you and you are unique, one of a kind. One of the ways to get free from finding your identity in what others think and say of you, is to know in your heart (not just intellectually) that you have a spiritual father or higher power that will always approve of you. This is all about getting connected to your spiritual source.

**What am I doing here?** If your answer is that your God created you to reach out to others in love, acceptance and forgiveness, then it means you have something worthwhile to live for, you have purpose, focus and direction for your life. This position will add richness and meaning to your life.

**Where am I going?** If your answer is that you are going back to your God when you die, then it implies that you have a future and a hope. You have something bigger than yourself to live for.

**Listen and follow what your heart is telling you.** Not everyone will necessarily agree with the above statements. Everyone is free to have their own opinion. The above philosophy works very well as a way of understanding and coping with life. We are not trying to get people to become religious so that they end up following a set of rules, rather it is about challenging people to get in touch with their true inner feelings, so that they will make decisions that are right for them. In our society people are of course allowed to believe in and experience God as they so wish. It is all about personal choice. Only the individual can find out what works for him/her.

**RATIONALE:** People with a good sense of identity and purpose are able to cope more easily with life's problems because they tend to see the bigger picture.

# Giving meaning to life

**Where do I come from?**

**What am I doing here?**

**Where am I going?**

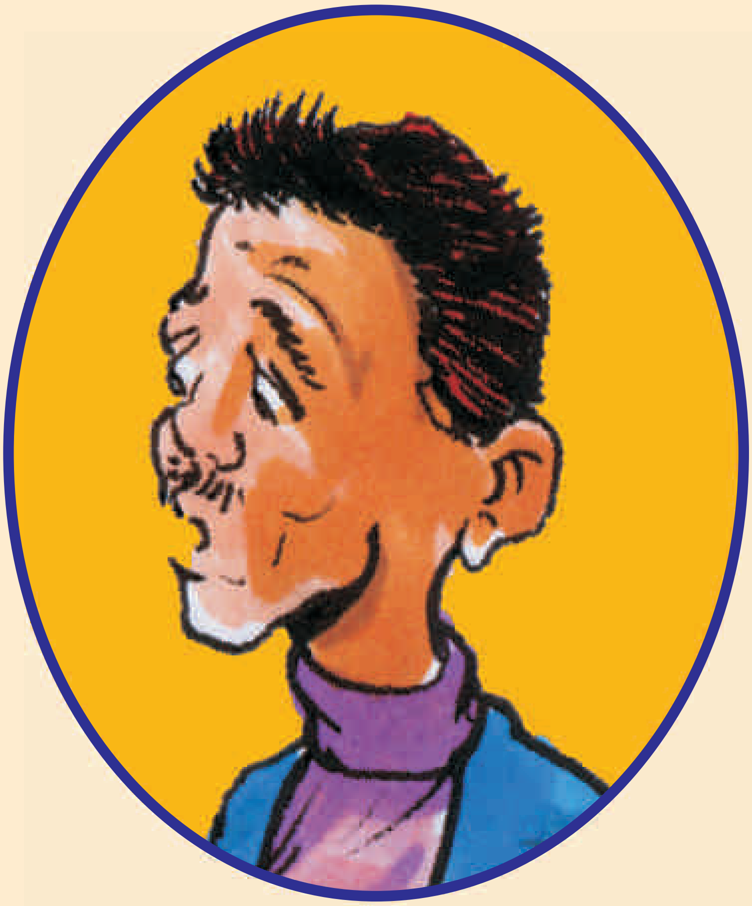

**Listen and follow what your heart is telling you**

## The storms of life will pass

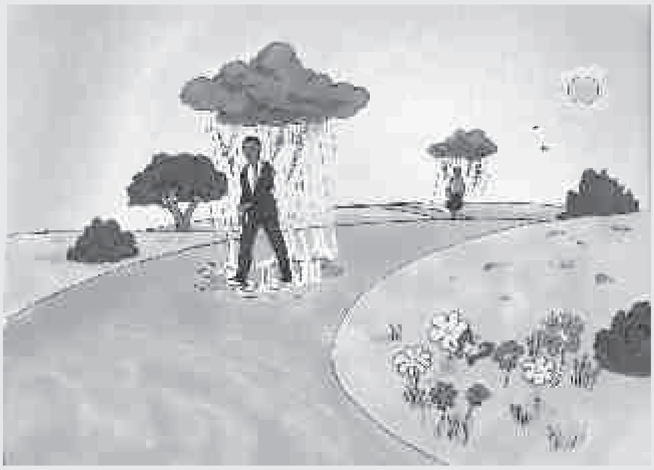

**Surviving storms make you stronger - you can cope**

**3d**

**AIM:** To reinforce the message of the previous poster, and to help our clients realise that learning to overcome the storms of life make you stronger.

**STRATEGY:** Use the graphic. There are 2 messages that can come out of this.

1. When you look at a storm from a distance, you can see that it is moving and will eventually pass. When you are in the middle of the storm all you see is the darkness, the rain, the hail and the lightning. You can become so pre-occupied with your problems that you do not realise that moving purposefully in the correct direction will get you out of the storm. If you run away from the storm i.e. life's problems, then the storm will follow you. If you turn into the storm i.e. deal with life's problems and overcome them, then you will get faster out of the storm.

2. We all go through the storms of life. At the time things seem terrible, but if we learn to overcome the problem then we realise that we are stronger because of the experience. If we learn to see life's difficulties as opportunities to grow, then all of the problems we face are given meaning in the greater scheme of things. What was previously seen as extremely negative, can be seen in a positive light.

   **Surviving storms make you stronger. You can cope because your God is in control.**
   Many of us feel isolated and alienated in life. In this situation it is easy to feel sorry for yourself and get depressed with all the bad things that are happening to you. When you get connected with your maker or your spiritual source you no longer need to feel alone in life.

**RATIONALE:** Your ability to deal with something depends on your perspective of it.

# The storms of life will pass

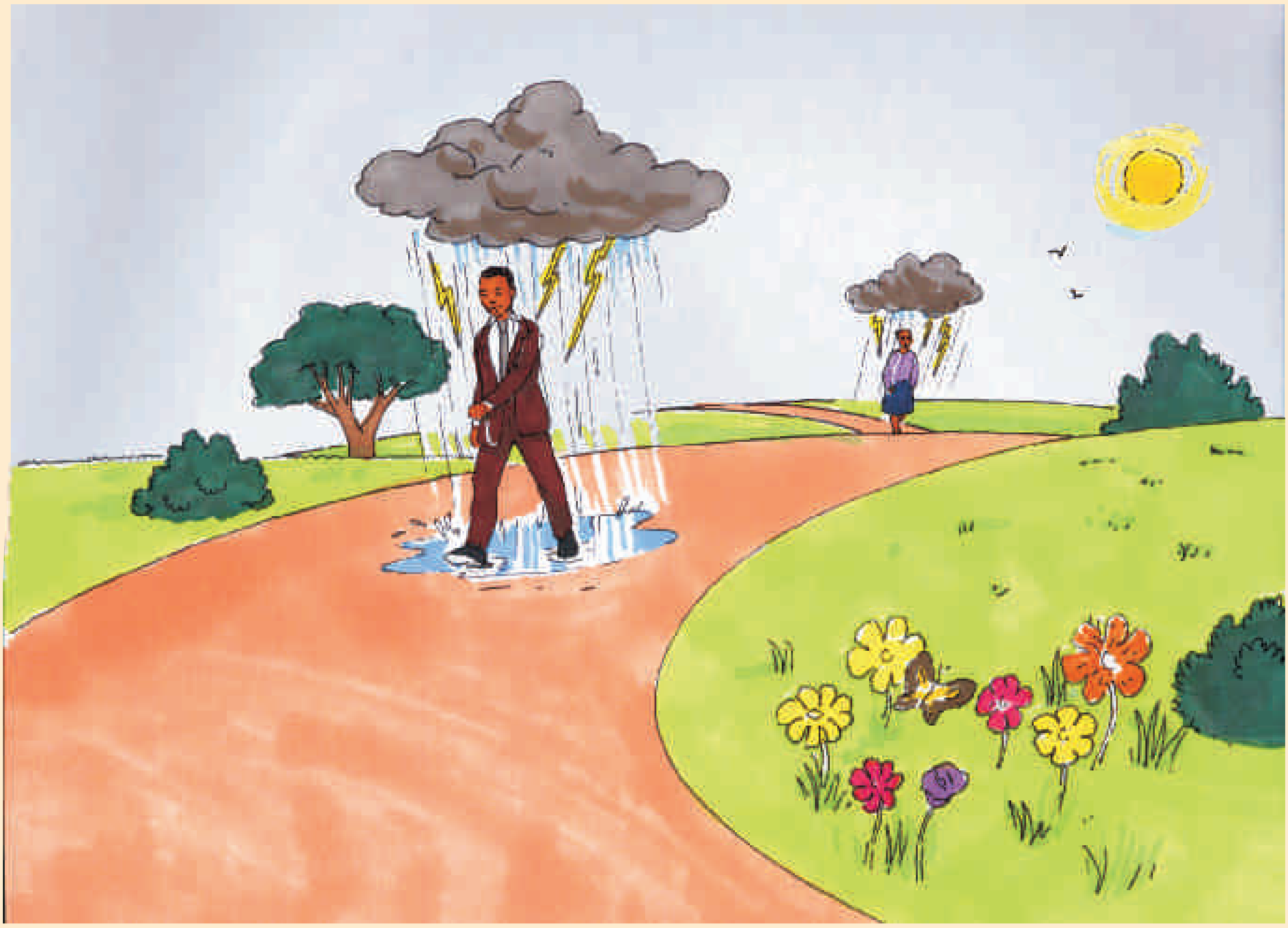

**Surviving storms make you stronger - you can cope**

**Someone might call you bad/stupid/ugly - that's just their opinion**

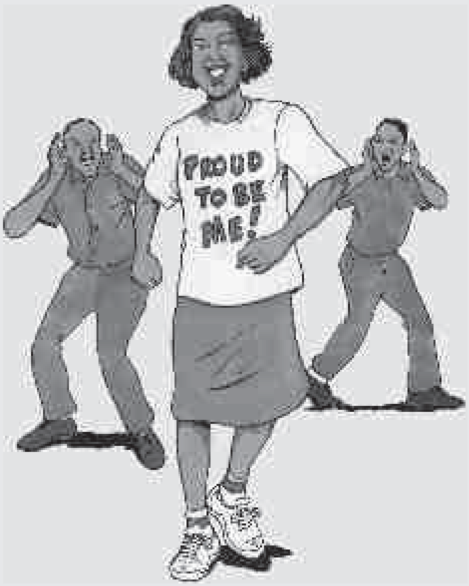


**You have value - no matter what people say about you!**

**3e**

**AIM:** To make our clients understand that every human being has inherent value because they are created by their God.

**STRATEGY:** This poster is a follow on from poster 3b. Because God or a higher power has created us all, one person is not better than another. We all have inherent value, irrespective of what people say about us. Use the graphic to illustrate this point. In life, many of us find our identity and value in what other people say about us. This can lead to us performing so that others can think better of us. To achieve this, we will even do things that don't really suit us, just to please someone else. This can make us very unhappy.

Encourage the clients to stand up as human beings with equal rights to anyone else, irrespective of title, position or power. No one should allow themselves to be walked over as if they were a doormat, or allow themselves to be belittled by others. The oppressor often tries to make you feel small so that they can feel good about themselves. This arises out of their own insecurity. The person with a solid sense of identity is in a good position to reach out to the oppressor, because it is the oppressor that is in need of help!

**RATIONALE:** You can only be victimized in life if you are willing to take up the victim role. If you stand up to the oppressor and refuse to be bullied, but demand to be treated with respect, then you can no longer be oppressed.

# Someone might call you bad/stupid/ugly - that's just their opinion

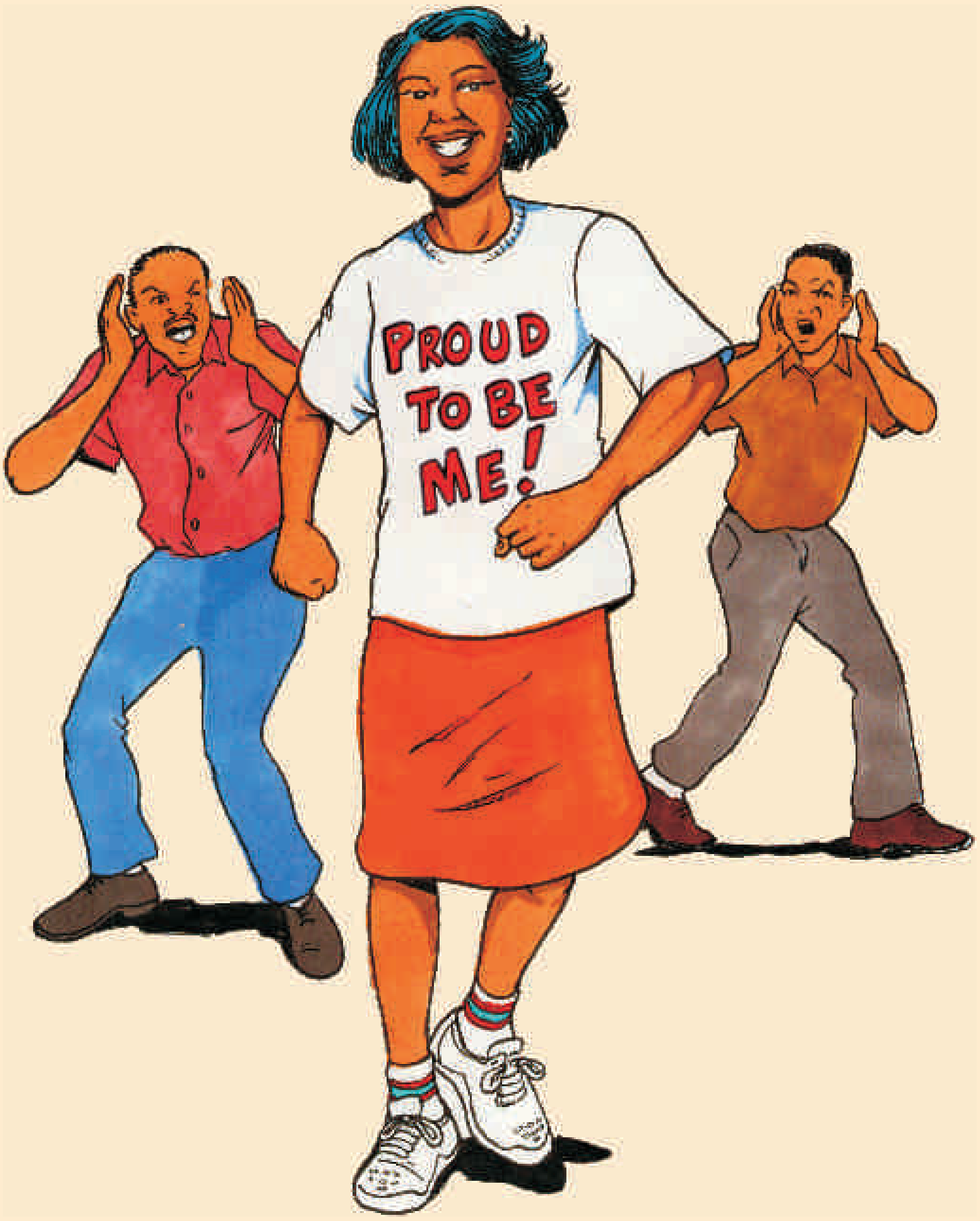


**You have value - no matter what people say about you!**

**What would you like others to say about you at your funeral?**

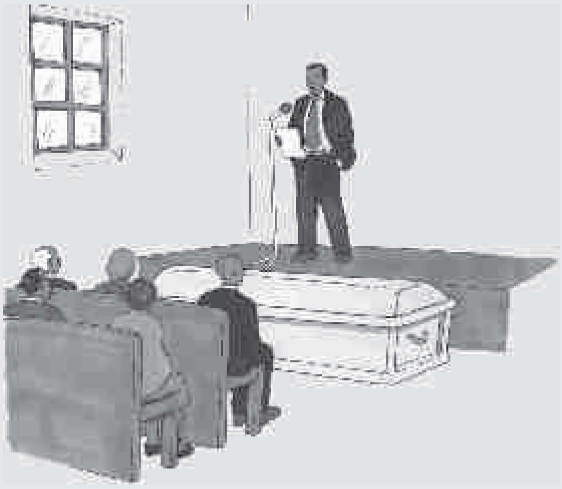

**Live your values**

**3f**

**AIM:** To take our clients through a meditation and visualisation exercise so that they can determine what their values are.

**STRATEGY:** Start with getting our clients relaxed and focussed with slow abdominal breathing and eyes closed.

**Visualize.** When they are ready ask them to see themselves attending a funeral. As they approach from the back they notice that there are already a lot of people sitting and listening to the speaker up front. Next to the speaker is the coffin. Get them to walk up to the coffin and realise with a shock that they are attending their own funeral. Then get them to sit down and listen to the speakers - a spouse, a child, a long time friend, a work colleague, a religious leader. What would they like these speakers to say about them? Were they good parents full of love, were they generous, caring people or did they not care about others? What should come out of this are the true values by which people would like to live their lives.

**Associate with others.** There is a good Afrikaans saying, "Jy word altyd soos jou vriende." In English a similar saying is, "You know someone by the company they keep." These are usually used in the negative sense e.g. bad company corrupts good character. However, the opposite is also true. If you would like to develop good values then associate with other people who are living those values and they will come easily to you.

**Work hard.** Nothing in life happens unless you decide you are going to do something about it. If you put in the effort then you will get what you want.

**Live your values.** See Rationale.

**RATIONALE:** If you live your values then everything you do in life will always have meaning for you.

# What would you like others to say about you at your funeral?

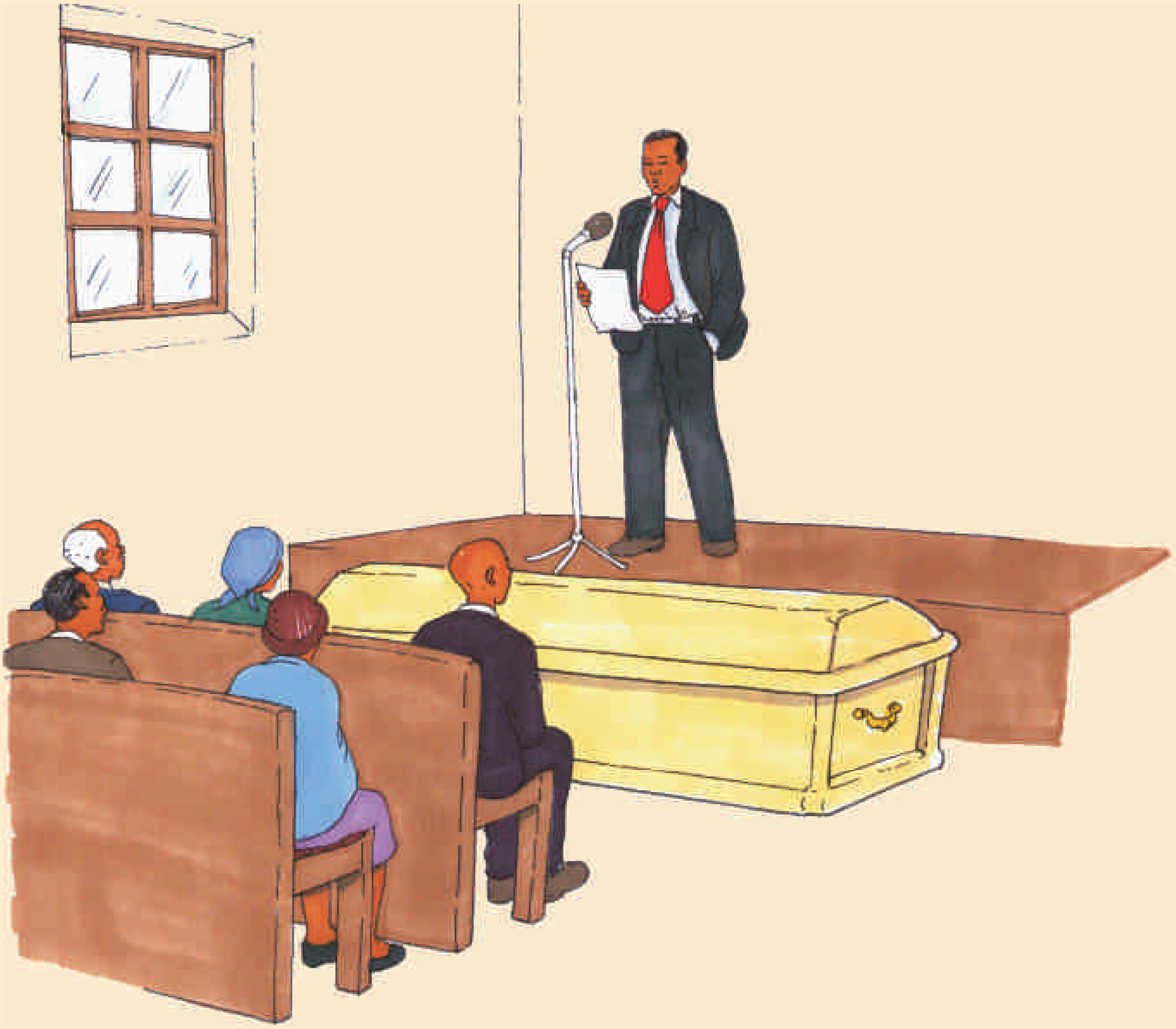

## Live your values

## See life's problems as learning opportunities

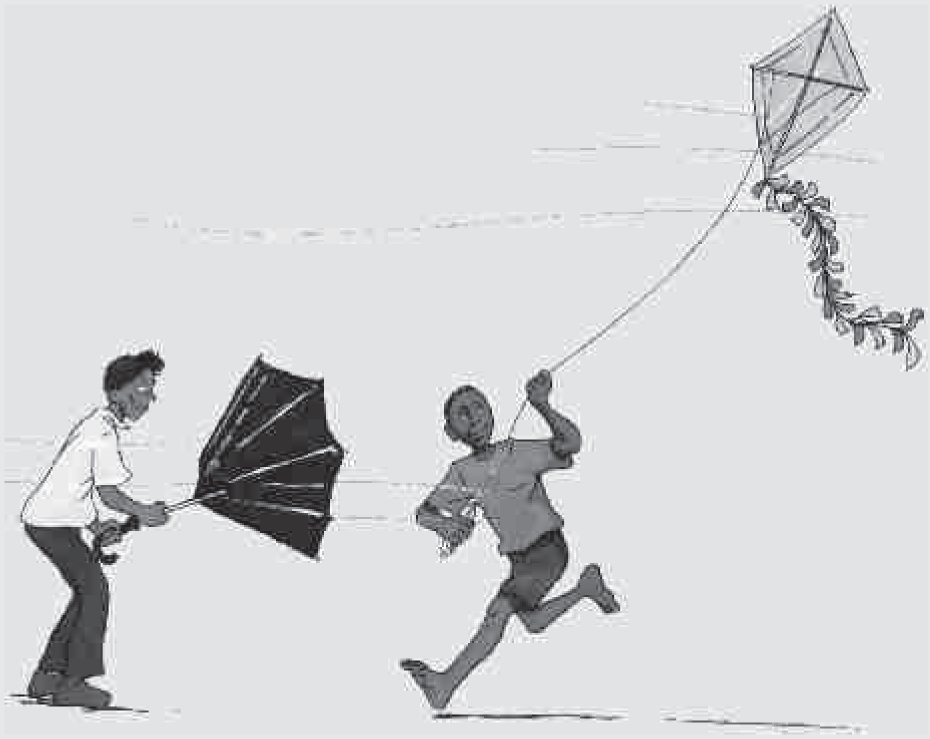

4a

# PART 4

**AIM:** To get our clients to approach life's problems from a different perspective and see them instead as learning opportunities.

**STRATEGY: E**xplain the graphic. Many of us have sat inside on a windy day and moaned about the weather. Like many other things in life, it is beyond our control, so we cannot change it. All we can change is the way we see things. Instead of seeing the wind as a huge problem, it can be seen as the perfect opportunity to fly a kite. We can fight against what we can't control and get incredibly frustrated, or we can use the conditions to our advantage.

**RATIONALE:** When we see life's problems as learning opportunities then life becomes a lot more bearable.

# See life's problems as learning opportunities

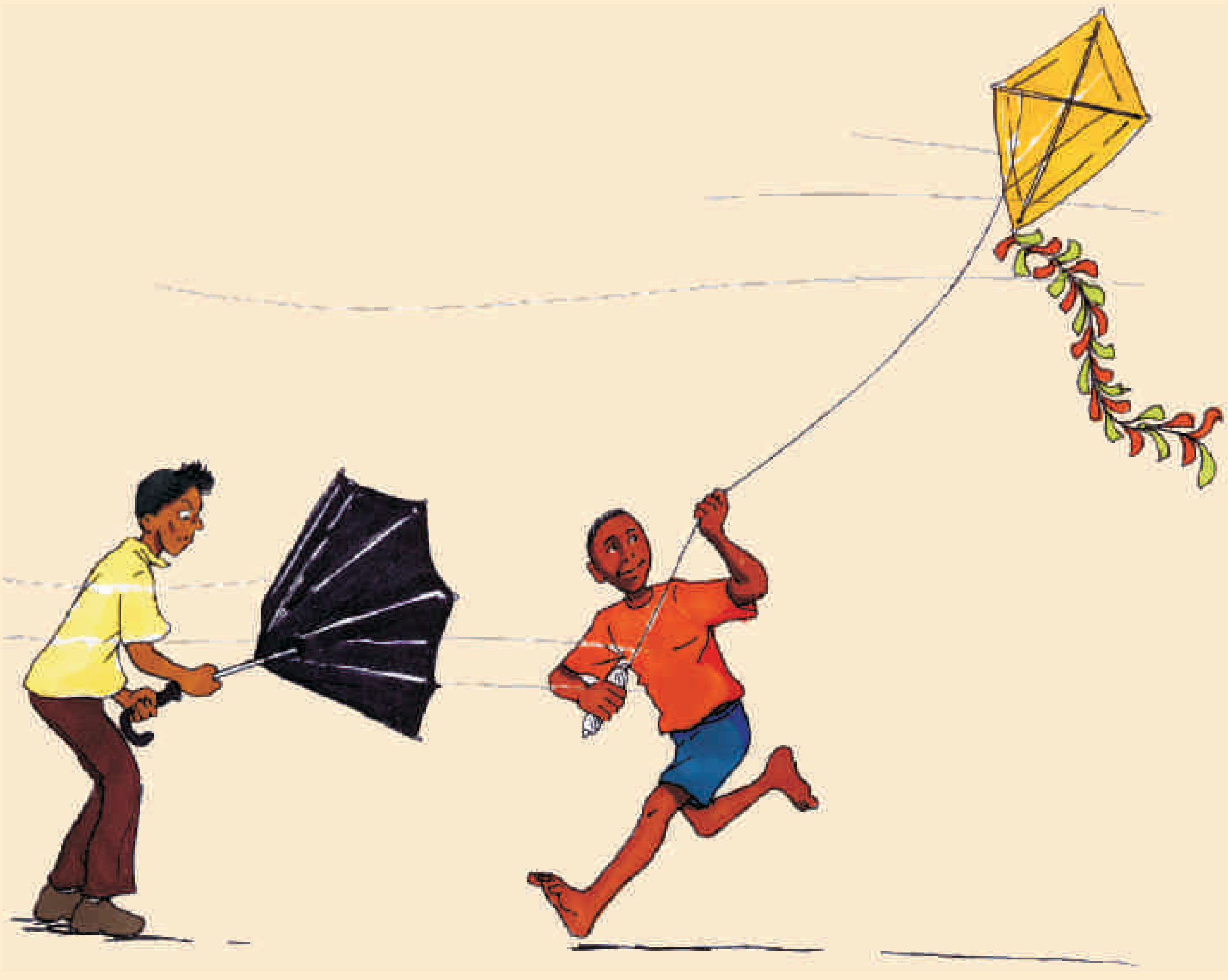

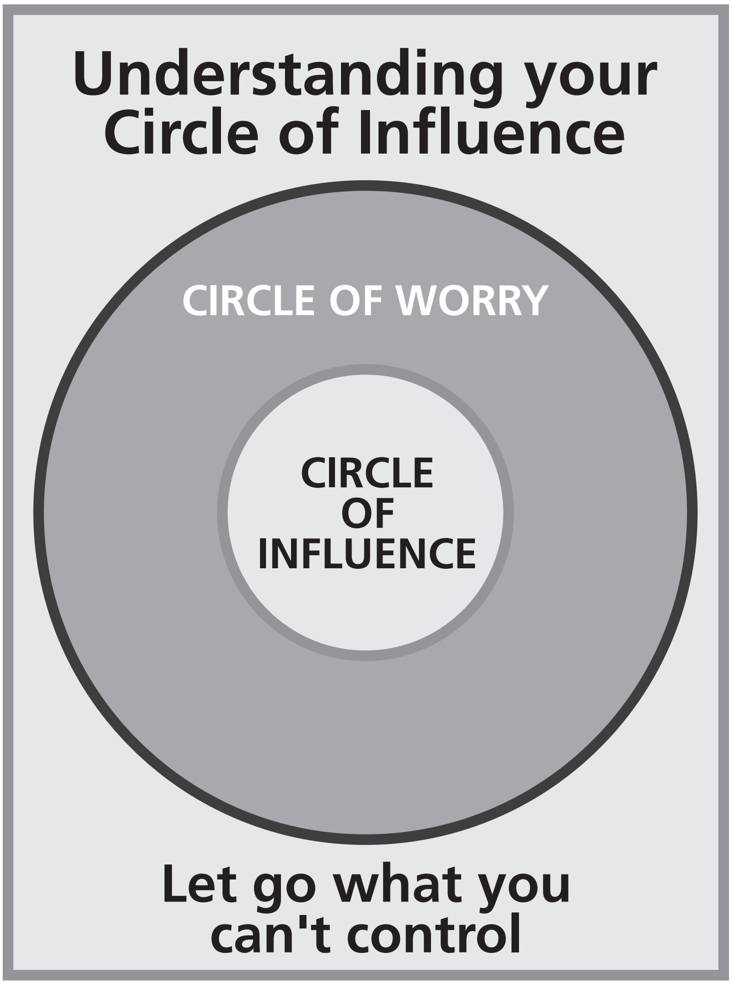


4b

**AIM:** To save our clients a lot of frustration by teaching them not to worry about things that are outside of their control.

**STRATEGY:** Explain the graphic. We all worry about lots of things. Often the things we worry about are things that other people are doing. If these people are adults, then they have made the choice to do what they are doing. All we can do is give our opinion because this is within our circle of influence and therefore under our control. The decision the other person takes is under his/her control and therefore outside our circle of influence. One cannot control another adult, so why worry about it.

Ask the clients for examples of this in their lives. Classic examples in our communities which you can discuss are:

- The suffering wife of an alcoholic husband;
- The suffering mother whose adult children are still living at home, but not willing to listen to her.

**Let go what you can't control.**

**RATIONALE:** Worrying about things you cannot change is a waste of energy and will result in you getting sick.

# Understanding your Circle of Influence

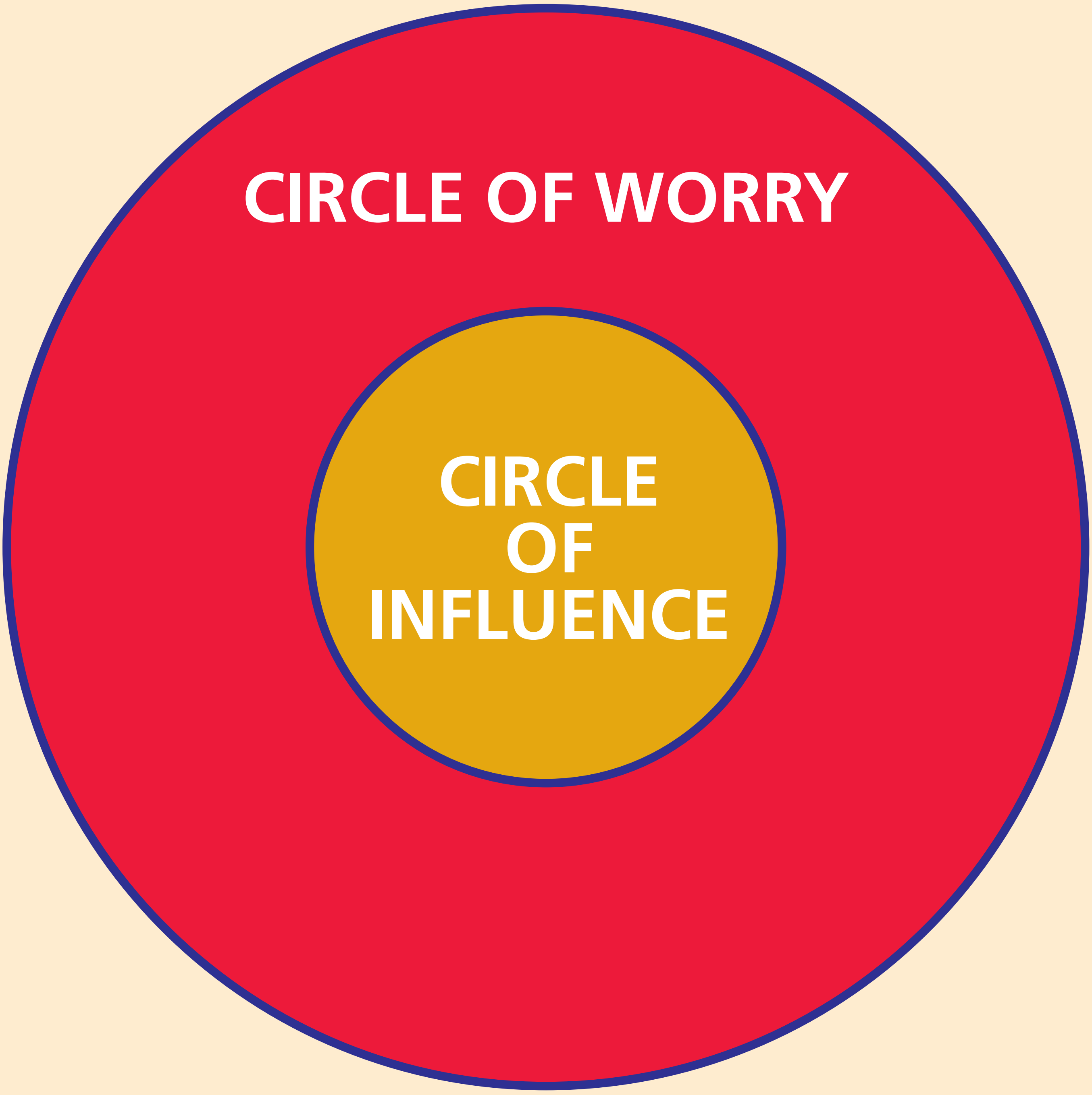


**Let go what you can't control**

# Why do some people hurt other people?

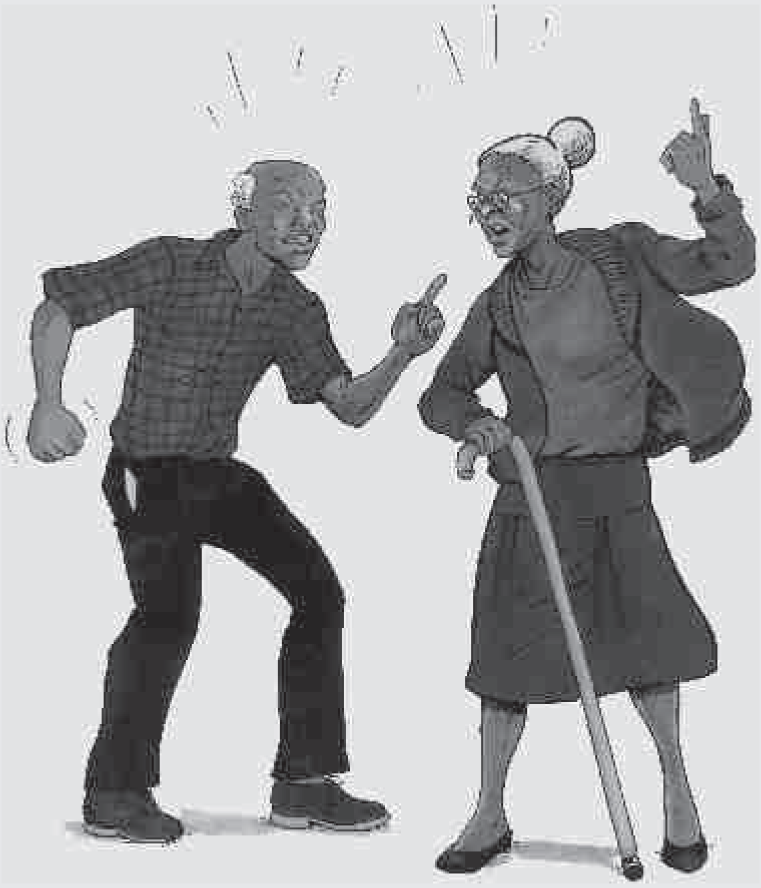

**4c**

**AIM:** To allow our clients to gain a different perspective on arguments and fights. This will in many cases allow them to resolve the situation.

**STRATEGY:** Using the graphic ask the question, "Why do some people hurt other people?"
Many would answer this question by describing them as bad, selfish or rude etc. The answer is because they themselves are insecure and hurt inside and in many cases are just expressing their frustrations and anger. With this new perspective, one can deal better with conflict situations. Instead of just fighting with the person, one can understand them as needing emotional help. If you reach out to the inner child in that person, then the problem can sometimes be quickly solved.

**RATIONALE:** One can perpetuate a fight by holding onto existing paradigms, or one can gain a new perspective and resolve the issue.

# Why do some people hurt other people?

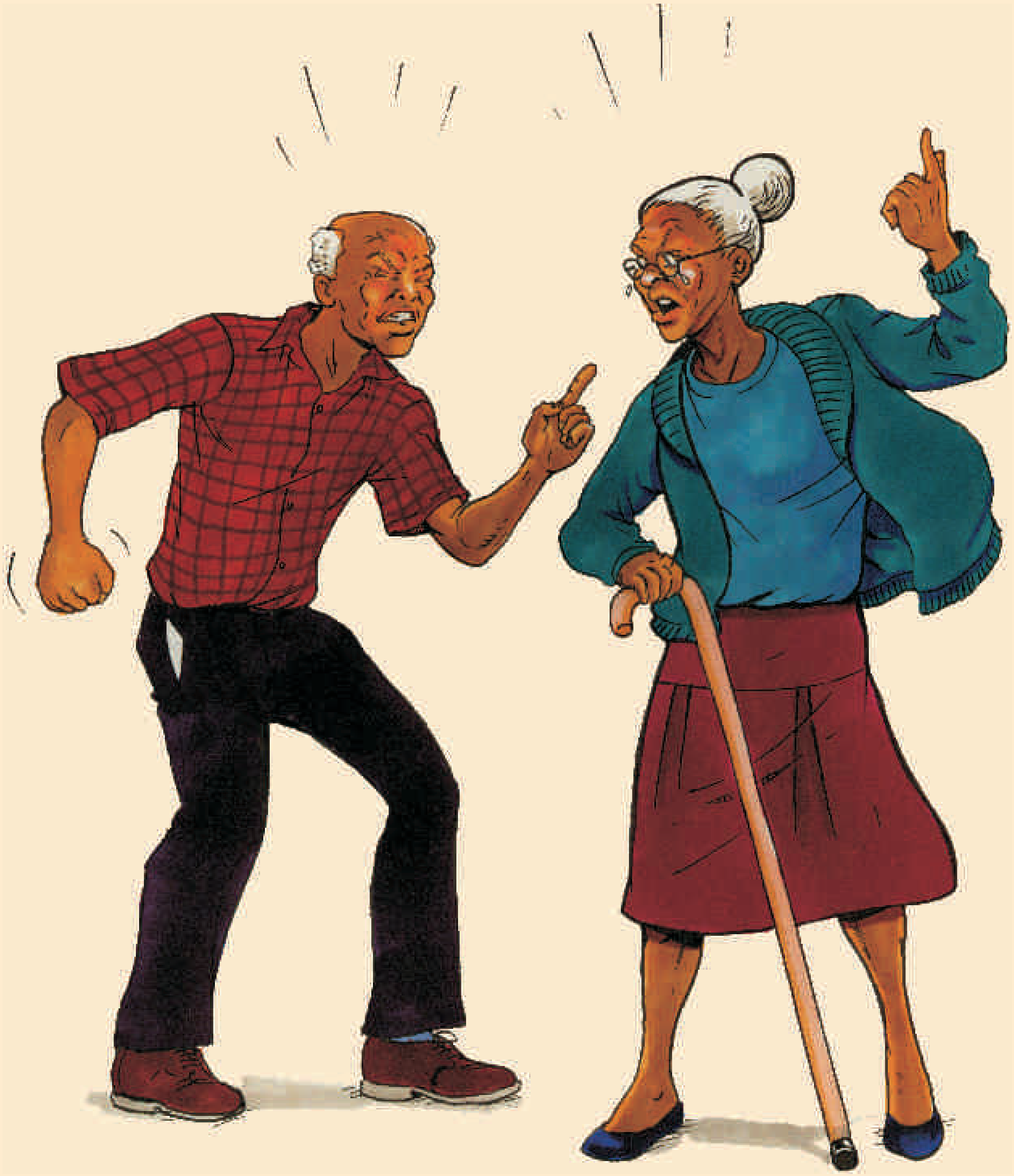

**AIM:** To teach our clients how to resolve traumatic issues from the past by using\meditation and visualisation techniques.

**STRATEGY:** Start by asking everyone to close their eyes and visualise a traumatic experience from the past e.g. a fight, an argument, a car accident etc. Ask everyone to open their eyes and tell you what they felt. When you think about a traumatic experience you will notice that you might feel tense or your breathing might become shallow, almost as if you were experiencing the trauma again. Unless you learn to let go of a situation, you can experience it a thousand times over, which can contribute to you getting sick. Tell the clients that we are going to teach them how to let go or dissociate from these bad memories.

**You may have to resolve issues in your life before you can move on**

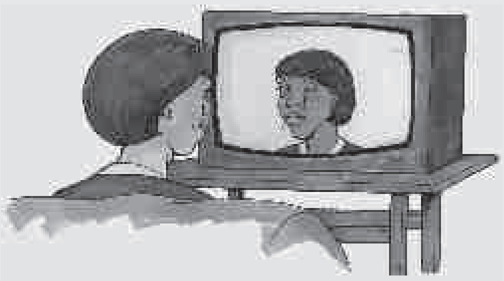

**Separate yourself from feelings**
**Try to forgive and understand:**

- **The situation**
- **Yourself**
- **The other person(s)**

**4d**

Get everyone relaxed through abdominal breathing and meditation with their eyes closed. When they are ready, ask them to imagine that they are watching a big TV screen. On the screen is playing the traumatic experience they recalled earlier, except this time they not involved in the situation, they are watching it as an interested yet uninvolved spectator. Tell the clients that if they are not able to emotionally distance themselves because the situation is too painful, then they can just stop the movie. Then ask them to focus on a situation where they felt happy or safe and breathe these feelings into the heart area. When settled they can restart the movie.

While they are watching the situation on the TV screen, <u>what can they learn</u> <u>from it</u> in terms of the <u>actual situation, themselves</u> and the <u>other person</u>. Ask them to imagine that the camera is changing angles and viewing the situation from another perspective. <u>Can they learn anything about the situation from this new angle?</u> Ask the clients if they need to forgive the <u>situation, themselves or the other person/s.</u>

**It's time to move on with life.** When they have understood and forgiven, ask the clients to make a conscious decision that what happened is in the past and they no longer have control over it. They can now release the situation into their God's hands and move on with their life.

**RATIONALE:** Traumatic experiences can damage our health unless they are resolved.

# You may have to resolve issues in your life before you can move on

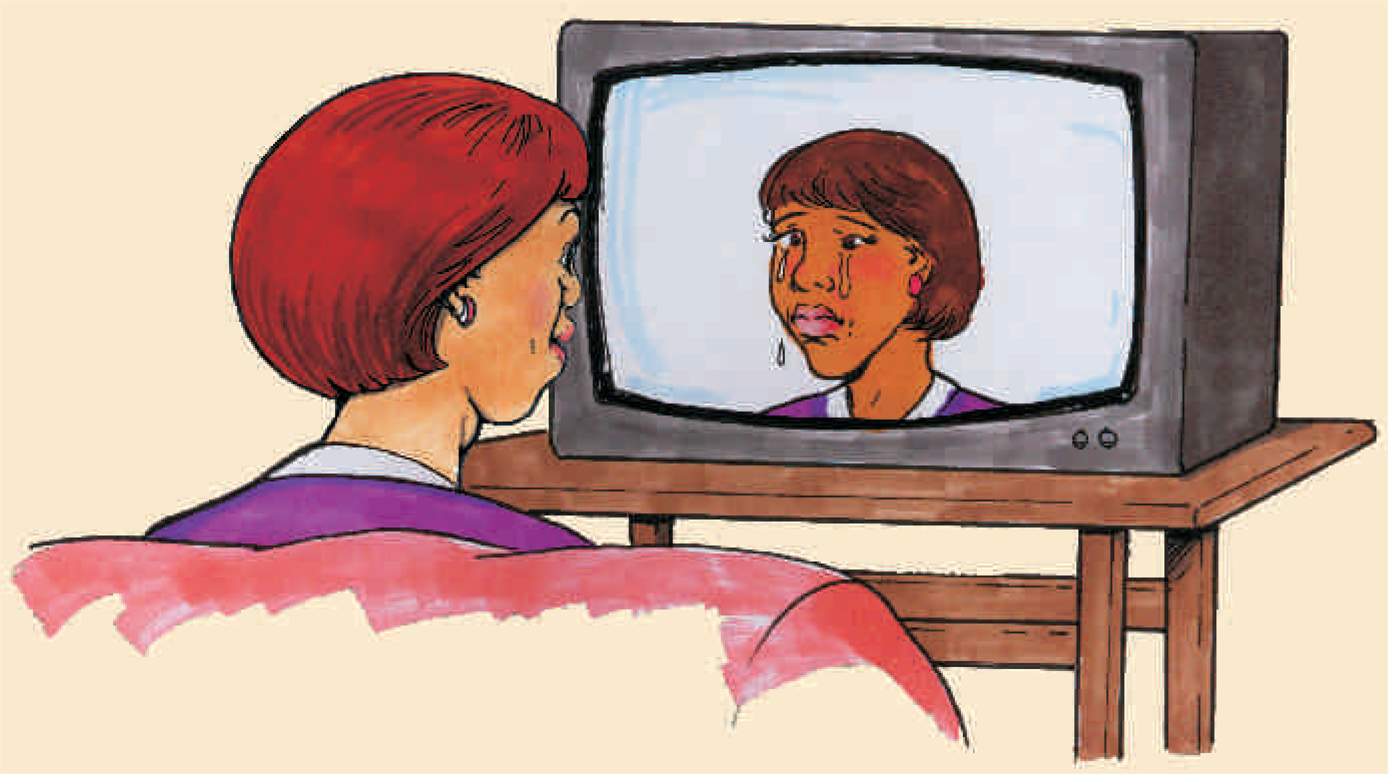

**Separate yourself from feelings**
**Try to forgive and understand:**

- The situation
- Yourself
- The other person(s)

# PART 5

**Are your negative emotions making you ill?**

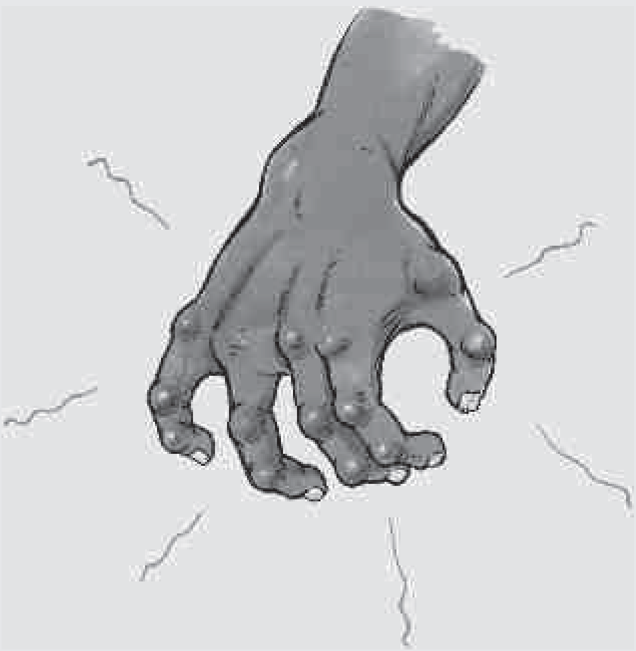

5a

**AIM:** To get our clients to make the link between their physical sickness and their emotional 'sickness'.

**STRATEGY:** You will need to explain this poster.

Point 1: We are all composed of a body, mind and a spirit - get agreement from the group. It's fine if someone does not agree as everyone has a right to their own opinion.

Point 2: The body, mind and spirit are one, they are inseparable. If your soul and spirit depart from your body then you are dead! - get agreement from the group.

Point 3: Continue with the argument. It therefore stands to reason that if you have a chronic sickness in your body, then this could reflect problems in your spirit e.g. arthritic pain could mean that there is underlying emotional pain or hurt - get agreement from the group.

Point 4: Therefore, to effectively deal with external pain, one must also deal with the internal pain. But how can you do this if you deny that the problem exists?

Point 5: **PLEASE EXPLAIN TO THE CLIENTS** - There are many different causes of disease included inherited, environmental, bacterial etc. - but negative emotions can cause or contribute to disease. Only the clients will know if this is true for themselves.

**RATIONALE:** To bring about lasting change one needs to understand and deal with the real situation, which is usually internal. Often, all we do is treat the external symptoms of the real problem with tablets, while the real problem gets worse.

# Are your negative emotions making you ill?

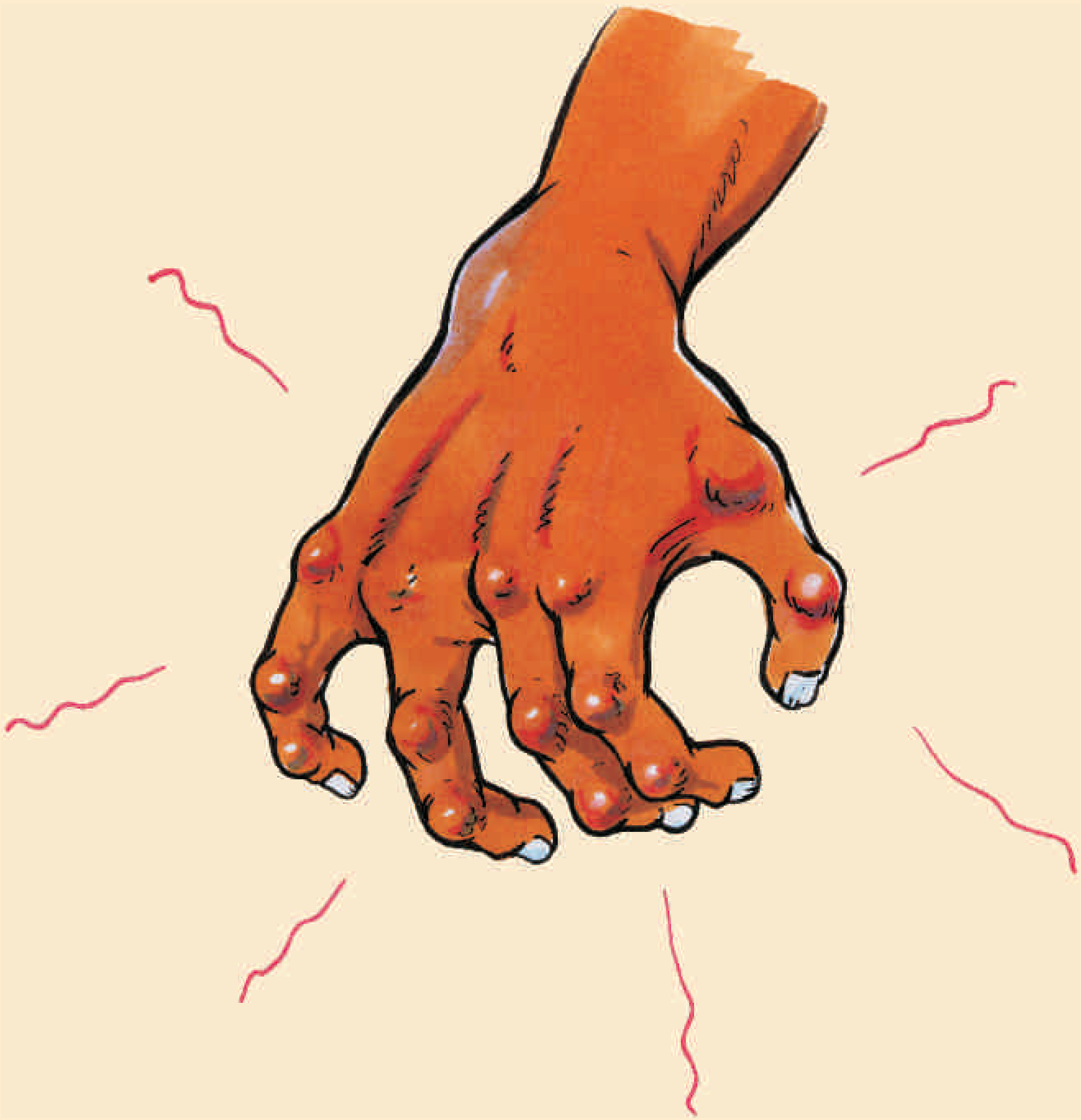

**AIM:** To help our clients understand the psychology of addiction and reinforce the link between what happens externally with the internal.

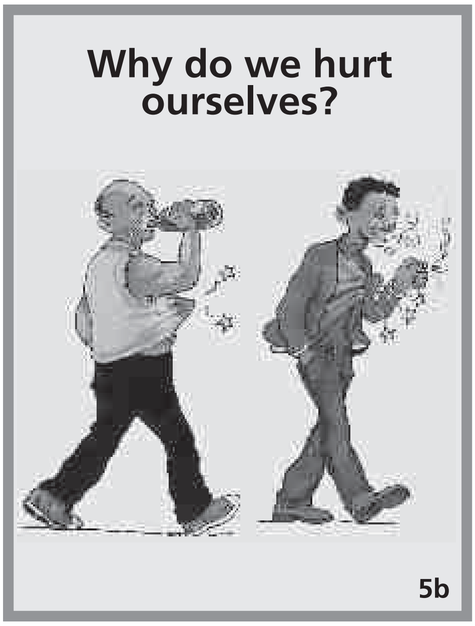


**STRATEGY:** Using the graphic ask the clients why people drink, smoke or take drugs. After all, they are not stupid, but they know they are doing something that is bad for their health.

**Q:** So why are they doing it?

**A:** We only do things because they work for us. e.g. Alcoholics drink because they are trying to numb the emotional pain they have inside them. Unfortunately it only deadens the pain for a short while, and when they sober up the pain is worse.

The emotional pain can be due to guilt, hurt, frustration, shame etc. Using alcohol is just a way of coping with the internal pain e.g. Heavy smokers and drug users use cigarettes to diffuse their internal tension. It helps them to relieve their stress. Therefore smoking or using drugs is just an external manifestation of an internal tension.

**Note to the presenters:** Getting people to stop smoking, drinking and using drugs can have unexpected side-effects. If someone stops smoking, but does not deal with the internal tension that is causing them to smoke, then they no longer have a way of releasing this tension. This internal tension can then manifest in different ways e.g. as a stomach ulcer, arthritis or pain, or might lead to spouse abuse, gambling etc. To truly resolve the issue, one has to deal with the root internal cause. Our clients would in many cases rather focus on the external problem, smoking, because dealing with the real issue can sometimes be too painful.

Secondly, you need to be aware that each family has a special dynamic, which is often in balance despite one of the members being dysfunctional. If a father is a dysfunctional alcoholic, other members of the family will take up part of the father's role and often enable him to continue with the abuse. If the father stops drinking then sometimes the family will break up, because the father has now changed roles, but the others are not willing to change their roles e.g. the mother might have benefitted from having the husband dependent on her and is not willing to change her role. If you find yourself in this situation always remember to involve the whole family.

**RATIONALE:** If you understand the real problem then you can deal with it or be referred for special help.

# Why do we hurt ourselves?

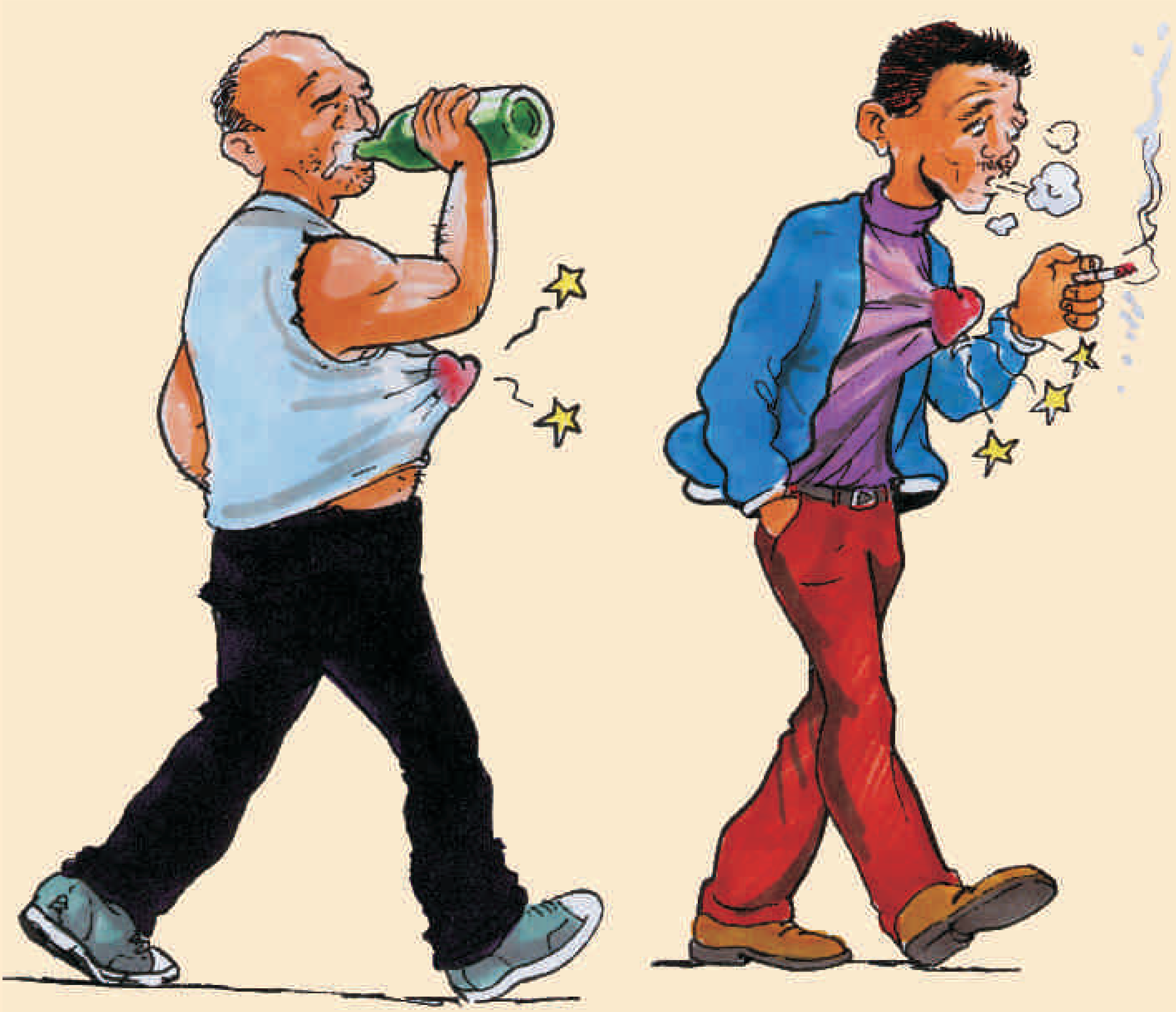

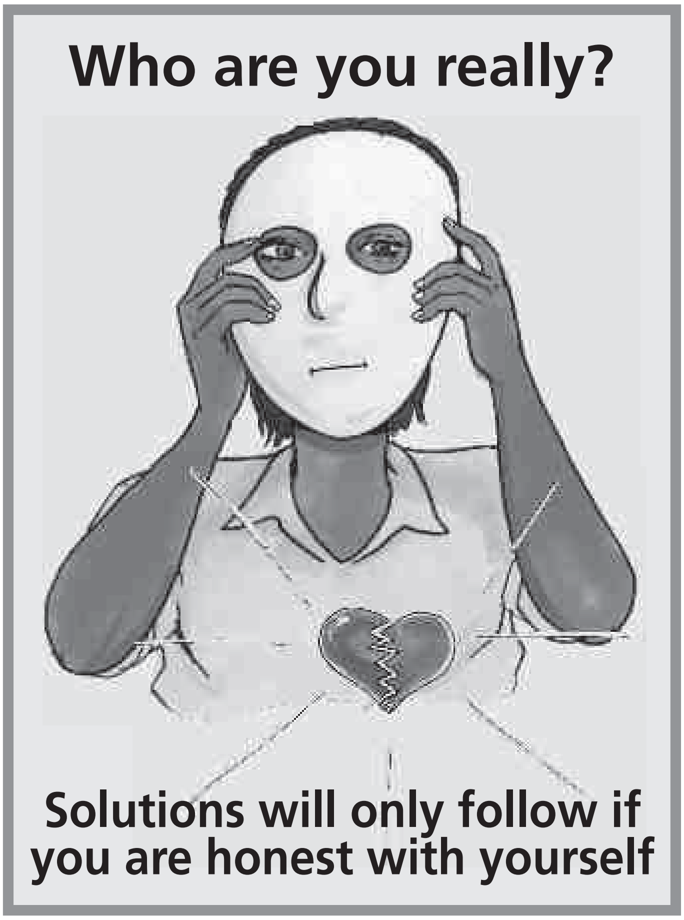

5c

**AIM:** To encourage our clients to get in touch with their true feelings and do something about it if there are problems.

**STRATEGY:** You will have to explain this.

We all hide our true feelings away behind 'masks'. We like to show to the world that everything is under control, when in reality our feelings are in turmoil.

Unless we accept who we truly are, we cannot make any meaningful change. We often avoid our true feelings because they hurt too much, but unless we confront these feelings and resolve them, we will always feel hurt inside.

We are all adults and have choices to make. We can continue to try and deal with the external symptoms of our internal pains and continue walking in pain or we can confront our true feelings and deal with them and discover our healing.

**Solutions will only follow if you are honest with yourself.**

**RATIONALE:** You can only resolve a problem in the long term if you deal with the root cause of the problem.

# Who are you really?

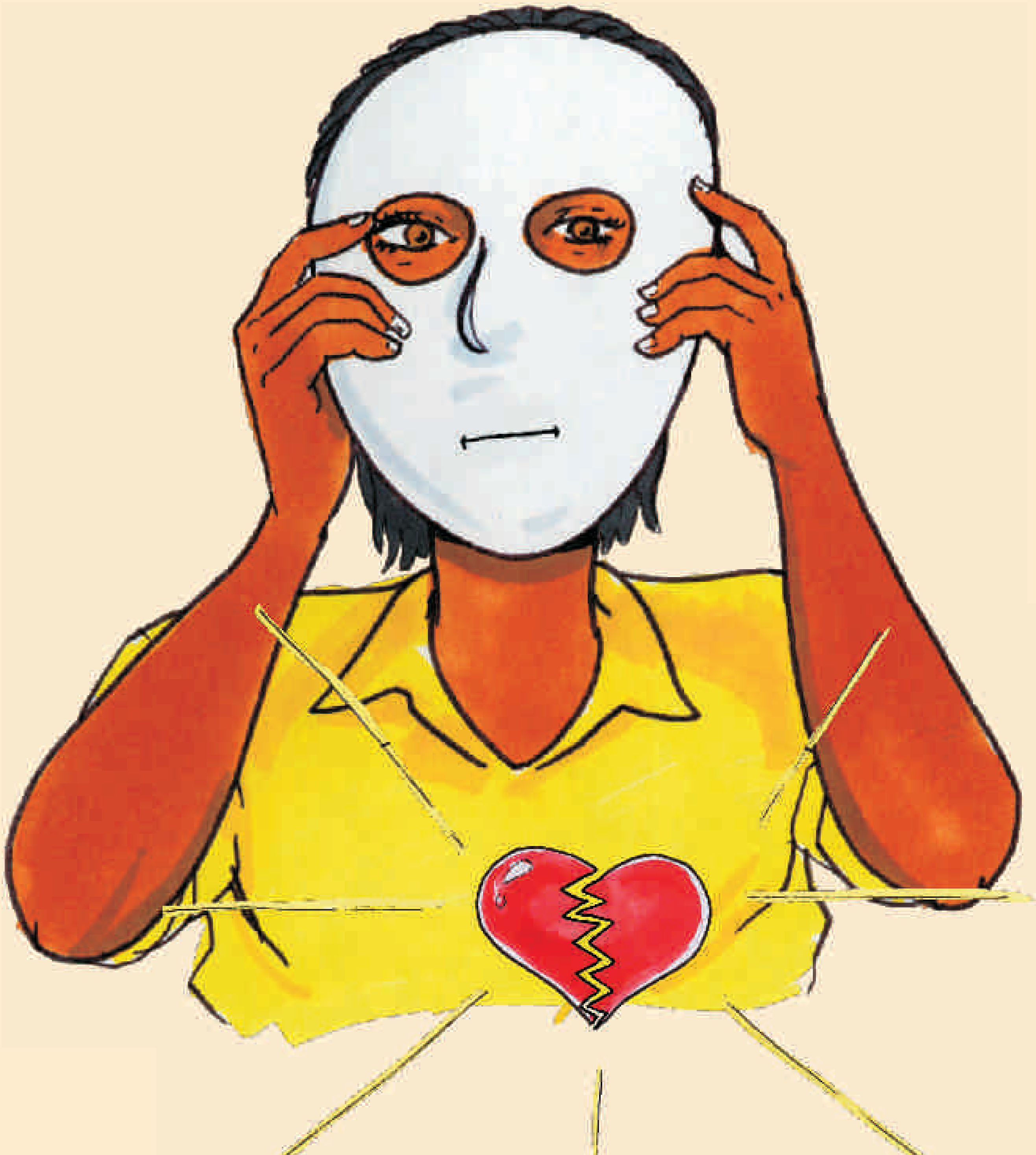

**Solutions will only follow if you are honest with yourself**

**AIM:** To model a possible path to healing for our clients.

**STRATEGY: Understand your real situation.** This was dealt with in the last 3 posters.

**The path to healing is a process**

- Understand your real situation
- Take responsibility
- Confront the situation with your God's help or with someone you can trust
- Accept and deal with your emotions
- Develop new habits - resist the old
- Adopt a thankful attitude
- Help others

5d

**Take responsibility.** Ask the clients, "Who is going to sort out their problems?" Of course, problems of this nature can only be sorted out if the client is willing to take ownership and responsibility of the problem.

**Confront the situation with your God's help or with someone you can trust.** Many of our clients will have tried in the past to sort out their own problems. Some might have succeeded but many others will have failed, which is one of the reasons why they are ill. To overcome their problems they will need help. This help can come from another person whom they can trust and confide in, but often because it is a spiritual problem they will need to go back to their God as discussed in poster 3c.

**Validate the feelings and emotions.** It is important to realise that our feelings are real. We do not have to feel ashamed of them or deny that they exist. If we feel hurt or lonely then that is just who we are. We do not have to feel as if we are inferior, because we are not our feelings. Only after we accept our feelings as they are, can we start to change them.

**Develop new habits and resist the old.** To change a habit takes a conscious decision to stop doing it and replace it with something else. Just trying to stop something is a negative way of approaching things and can be difficult to achieve. When you put a lot of focus on the new habit and start seeing the benefits, then the old habit automatically falls away.

**Thankful attitude.** Being thankful for what you do have and not putting all your focus on what you do not have, is a pre-requisite for being content with life.

**Help others.** In life you have to give to receive. As you learn new habits it is good to use your new skills to help others. As you do this the new habits become more ingrained in your life, so that by helping others you actually end up helping yourself.

**Remember: this is a journey!** Remember to constantly remind our clients that lasting change is a process that can take years or even a lifetime to accomplish. As long as they know what they want to achieve and constantly work at it, they will achieve their goal.

# The path to healing is a process

- Understand your real situation
- Take responsibility
- Confront the situation with your God's help or with someone you can trust
- Accept and deal with your emotions
- Develop new habits - resist the old
- Adopt a thankful attitude
- Help others

# Acknowledgements

**The development of the flipchart is a joint collaboration between the Metro District Health Services (MDHS) of the Provincial Government of the Western Cape (PGWC) and the City of Cape Town (CoCT).**

**Concept:**
- Dr Andy Young: City of Cape Town.

**Project Development:**
- Maureen Mc Crea: Health Promotion, MDHS
- Unita van Vuuren: Chronic Disease Management, MDHS
- Graham Clark: Occupational Therapy, MDHS
- Priscilla Beeton: Health Resource Centre, City of Cape Town
- Hazel Bradley: School of Public Health, UWC

**Pictures:**
- Saeed Rhabeni: private artist

**Design:**
- Helene de Villiers: Health Resource Centre, City of Cape Town

**Pre-testing:**
- Lifetyle Change Team: Elsies River CHC

**Piloting and testing:**
- The Media and Training Centre, sponsored by MDHS: Community Outreach

**Printing of flipchart sponsorship**
- MDHS: Community Outreach Fund
- MDHS: Hospital Management Quality Improvement Grant.

**Production**
- Video and Media Production cc (VAMP)
